# Incidence and Mortality of Prostate Cancer in Canada during 1992–2010

**DOI:** 10.3390/curroncol28010096

**Published:** 2021-02-21

**Authors:** François Lagacé, Feras M. Ghazawi, Michelle Le, Evgeny Savin, Andrei Zubarev, Mathieu Powell, Linda Moreau, Denis Sasseville, Ioana Popa, Ivan V. Litvinov

**Affiliations:** 1 Department of Medicine, McGill University, Montreal, QC H4A 3J1, Canada; francois.lagace@mail.mcgill.ca (F.L.); michelle.le@mail.mcgill.ca (M.L.); mcgill_ca@outlook.com (E.S.); awz.research@gmail.com (A.Z.); mathieu.powell@mail.mcgill.ca (M.P.); linda.moreau@mcgill.ca (L.M.); denis.sasseville@mcgill.ca (D.S.); 2 Department of Medicine, University of Ottawa, Ottawa, ON K1N 6N5, Canada; feras.al-ghazawi@mail.mcgill.ca; 3 Division of Urologic Surgery, Université de Montréal, Montreal, QC H3C 3J7, Canada; ioana.popa@umontreal.ca

**Keywords:** prostate cancer, Canada, epidemiology, incidence, mortality

## Abstract

In Canada, prostate cancer is the most common reportable malignancy in men. We assessed the temporal trends of prostate cancer to gain insight into the geographic incidence and mortality trends of this disease. Three independent population-based cancer registries were used to retrospectively analyze demographic data on Canadian men diagnosed with prostate cancer and men who died of prostate cancer between the years of 1992 and 2010. The incidence and mortality rates were calculated at the provincial, city, and forward sortation area (FSA) postal code levels by using population counts that were obtained from the Canadian Census of Population. The Canadian average incidence rate was 113.57 cases per 100,000 males. There has been an overall increasing trend in crude prostate cancer incidence between 1992 and 2010 with three peaks, in 1993, 2001, and 2007. However, age-adjusted incidence rates showed no significant increase over time. The national mortality rate was calculated to be 24.13 deaths per 100,000 males per year. A decrease was noted in crude and age-adjusted mortality rates between 1992 and 2010. Several provinces, cities, and FSAs had higher incidence/mortality rates than the national average. Several of the FSA postal codes with the highest incidence/mortality rates were adjacent to one another. Several Canadian regions of high incidence for prostate cancer have been identified through this study and temporal trends are consistent with those reported in the literature. These results will serve as a foundation for future studies that will seek to identify new regional risk factors and etiologic agents.

## 1. Introduction

In Canada, prostate cancer is the most common reportable cancer and represents the third leading cause of cancer-related deaths among men. Studies have found that up to 80% of cases are diagnosed in men above the age of 65. As the number of men within this age group has been predicted to increase up to fourfold by the year 2050, this malignancy is becoming an important and growing public health concern [1].

Canada is a large multiethnic country covering approximately 10 million square kilometres with a population >37 million and an average population density of 3.6 individuals per square kilometer. Approximately 18% of the population lives in rural communities, which account for 95% of Canada’s surface area [2]. The remainder of the population lives in urban communities, which make up only 5% of the surface area. In particular, 60% of the population lives in metropolitan cities, with 1 in 3 Canadians living in either Toronto, Montreal, or Vancouver [2]. The Canadian health-care system consists of individual provincial and territorial health insurance plans, which are regulated by national standards established by the Canadian Health Act of 1984 [2]. There are an estimated 2.48 physicians per 1000 individuals, and approximately 47.2% of all physicians are general practitioners [2].

Prostate cancer screening guidelines exist at the national level; however, most individual provinces do not have asymptomatic screening programs for this malignancy [3]. To illustrate, the Canadian Task Force on Preventative Health Care and the Canadian Cancer Society published guidelines for asymptomatic prostate cancer screening in 1994 and 2000, respectively [3,4,5]. The former recommends not to order the prostate specific antigen (PSA) test for asymptomatic screening, whereas the latter recommends discussing its risks and benefits with a physician [3,4]. Despite these recommendation, in 2003, ~50% of men >50 reported undergoing a PSA test in their lifetime [3]. The widespread use of PSA screening in Canada has led to increasing rates of prostate cancer in the 1990s, mostly from overdiagnosis [5]. Overdiagnosis signifies the detection of a malignancy for which the natural course would not result in a significant morbidity or mortality [5]. Some studies report that, among the men, who received the diagnosis of prostate cancer, the prevalence of overdiagnosis was as high as 40% to 56% [5].

In addition, many risk factors have been linked to prostate cancer. Primary risk factors include advanced age, African–American ethnicity, obesity, and family history of the disease [6]. Men ≥65 are 17 times more likely to develop prostate cancer than those <65 [7]. Risks of developing a prostate cancer also increase 2–3 fold for individuals that have a first-degree relative (father, son, brother) with a prostate cancer [7]. Recent studies have also shown that the risk of prostate cancer in obese men is ~2 times higher than in non-obese men [7].

Other modifiable risk factors include diets that are high in meats, dairy, and fat; vitamin D deficiency in early age; increased levels of insulin-like growth factor 1; increased androgen levels; exposure to pesticides; and a history of a sexually transmitted disease [1,7,8]. The relationship between other risk factors such as vasectomy, sexual activity, smoking, alcohol consumption, occupation, social class, and prostate cancer still remains unclear [1,7]. Consumption of selenium (found in grains and fish) and vitamin E can decrease the risk of developing the disease [8]. Other research groups have shown that lycopene found in tomatoes, tomato products, pink grapefruit, and watermelon reduces the risk of prostate cancer [9].

The extensive list of modifiable risk factors for prostate cancer, which include potential environmental/occupational exposures, has prompted us to search for areas of geographic clustering for this malignancy and to analyze its temporal trends in Canada. To our knowledge, no research has attempted to map the incidence and mortality rates of prostate cancer at the city and forward sortation area (FSA) postal code levels in Canada.

## 2. Material and Methods

The CISS-RDC-668035 and the 13-SSH-MCG-3749-S001 protocols were approved by the Social Sciences and Humanities Research Council of Canada (SSHRC) and the Québec Inter-University Center for Social Statistics (QICSS), respectively. As per the institution policy, this study was exempted by the Research Ethics Board of McGill University.

### 2.1. Data Collection

The Canadian Cancer Registry (CCR) and Le Registre Québécois du Cancer (LRQC) are two distinct population-based cancer registries that contain demographic data on patients diagnosed with a primary malignant neoplasm. The demographic data include the patient’s sex, year of diagnosis, age at diagnosis, geographic location (province, city, forward sortation area), as well as the International Classification of Diseases for Oncology (ICD-O-3) code representing a given neoplasm. According to the SSHRC/QICSS guidelines, the forward sortation area (FSA), which represents the first 3 entries of a postal code, is the smallest unit that is allowed for geographical analysis. Incidence data were collected from the CCR for all Canadian provinces and territories outside of Quebec, and from the LRQC for the province of Quebec, as described in previous studies [10,11,12,13,14,15,16,17,18,19,20,21,22,23,24,25,26,27,28]. The CCR contains data from 1992 to 2015, whereas the LRQC only has the data from 1992 to 2010. We chose to analyze incidence rates for all provinces up until 2010. The Canadian Vital Statistics (CVS) is another database that contains data on all Canadian patients who have died from cancer. It was used to collect mortality data on prostate cancer-related deaths between the years 1992 and 2010. Information on the patient’s sex, year deceased, age at the time of death, location (FSA, city, and province), as well as the International Statistical Classification of Diseases and Related Health Problems (ICD) code for the neoplasm (cause of death) are included in this database. All three of these databases only include information on invasive cancers, and therefore, non-invasive cancers were not studied.

Data were retrieved from the CCR and LRQC using the tumor’s topography code, designating its primary site for prostate gland (code CD61.9) and for ICD-O-3 codes that are presented in Table 1. Similarly, mortality data were retrieved from the CVS by using the ICD-9 codes for the years 1992 to 1999 and the corresponding ICD-10 codes for the years 2000 to 2010. The Canadian Census of Population for the years 1996, 2001, 2006, and 2011 was used to obtain population counts by country, province, city, and FSA. We excluded cities and FSAs that had a male population <5000, as per the SSHRC regulations.

Quintile analyses were used to assess the relationship between prostate cancer incidence and socioeconomic status and ethnicity. For each FSA, the socioeconomic status (SES) and the percentage of African–Canadian/black individuals were calculated based on data from the Canadian Census of Population for the years 2001 and 2006. We used the median income per household as a surrogate for SES. For the SES analysis, FSAs were categorized into one of five quintiles (Q1_SES_ to Q5_SES_) according to its average median income. FSAs with the lowest average median income were placed in the first quintile (Q1_SES_) and those with the highest were placed in the fifth (Q5_SES_). Similarly, FSAs were categorized into a quintile (Q1 to Q5) based on the percentage of African–Canadian/Black individuals. Quintiles were compared to one another via incidence rate ratios (IRR) and their corresponding 95% confidence intervals (95% CI). Regression analyses were used to establish associations between provincial rates of prostate cancer and the provincial percentages of African–Canadian/Black individuals, the provincial percentages of individuals over the age of 65, and the provincial percentages of obese individuals. Correlations were considered statistically significant if *p* < 0.05.

### 2.2. Mandatory Data Rounding

SSHRC/Statistics Canada require researchers to round each frequency count to a multiple of 5 (lower or higher) via a random rounding scheme in order to respect patient confidentiality rules. In addition, frequency counts that are greater or equal to 1 and less than 5 cannot be released according to the SSHRC guidelines.

### 2.3. Data Analysis

Incidence/mortality rates and their corresponding 95% CIs were determined for each age group, year of diagnosis or death, province, city, and FSA. These rates are expressed per 100,000 males per year. The 95% CIs were calculated using the Poisson exact distribution and adjusted for rare events. The national age-adjusted incidence and mortality rates were calculated by the direct standardization method using the WHO 2000–2025 population as a standard. Age-standardized incidence and mortality rates by year, province, and FSA were calculated using the indirect method with the 2001 Census population as a standard. Trends over time were assessed using simple regression models and joinpoint regression analysis. The joinpoint regression analysis determines the best-fitting regression line and determines whether there are points in time (joinpoints) where significant changes take place. The ArcMap software was used for mapping and for geographical analysis.

## 3. Results

### 3.1. Demographic Information on Canadian Patients

Approximately 327,810 Canadian men were diagnosed with one of the 12 subtypes of prostate cancer listed in Table 1 between 1992 and 2010. These cases predominantly represented adenocarcinomas NOS (99.81%). Between 1992 and 2010, there were five cases of prostate cancer in patients ≤ 9 years of age; 70 cases in patients between ages 20 and 39; 55,165 cases (17%) in patients between ages 40 and 59; 233,685 cases (71%) in patients between ages 60 and 79, and 38,845 cases (12%) in men ≥ 80 (Supplementary Table S1). The mean ± SD age at the time of diagnosis was 68.5 ± 9.16.

### 3.2. Analysis of Incidence and Geographic Distribution of Cases of Prostate Cancer in Canada

**Figure 1:**
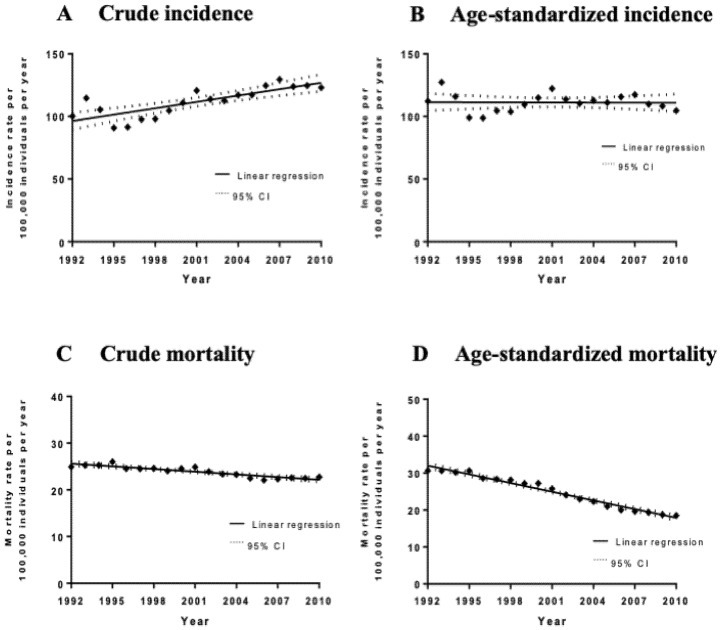
Incidence and mortality rates (per 100,000 individuals per year) of all cases and deaths between 1992 and 2010 with the line of best fit, and linear regression analysis of the incidence rate over time. (**A**) Crude incidence trends. The slope of the line is 1.67 ± 0.303 cases per 100,000 males per year, *p* < 0.0001. (**B**) Age-standardized incidence trends. The slope of the line is −0.028 ± 0.312 cases per 100,000 males per year, *p* = 0.93. (**C**) Crude mortality trends. The slope of the line is −0.19 ± 0.022 cases per 100,000 males per year, *p* < 0.0001. (**D**) Age-standardized mortality trends. The slope of the line is −0.79 ± 0.032 cases per 100,000 males per year, *p* < 0.0001.

The average age-adjusted Canadian incidence rate for the period of 1992–2010 was 112.84 cases per 100,000 men per year and the crude incidence rate was 113.57 cases per 100,000 men per year. Overall, there has been an increase in crude prostate cancer incidence/diagnosis rates over the 19-year period analyzed by 1.70 ± 0.30 cases per 100,000 males per year (*p* < 0.0001) (Figure 1). However, there was no significant increase in age-adjusted incidence rates during this time period (slope = −0.028 ± 0.31 cases per 100,000 males per year, *p* = 0.93). Notably, no significant joinpoints were identified via a joinpoint regression analysis of crude and age-adjusted incidence rates.

**Figure 2:**
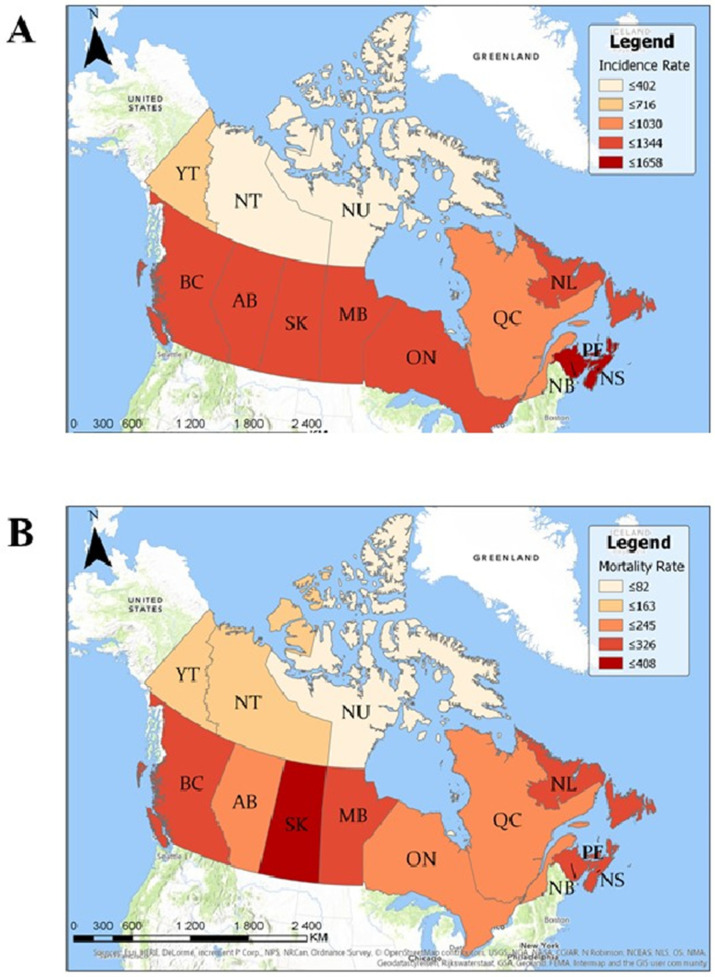
Geographic map of prostate cancer incidence and mortality rates per 1,000,000 men per year by province. (**A**) Rates of prostate cancer incidence per 1 million men per year by province between 1992 and 2010. Higher incidence rates are represented by darker shades of red/brown. (**B**) Mortality rates of prostate cancer per 1 million men per year by province between 1992 and 2010. Higher mortality rates are represented by darker shades of red/brown.

Seven provinces had statistically significant higher crude incidence/diagnosis rates than the national average: Prince Edward Island, New Brunswick, Nova Scotia, British Columbia, Saskatchewan, Manitoba, and Ontario (Table 2, Figure 2A). Seven provinces and territories had statistically significant lower crude incidence rates than the national average: Alberta, Québec, Yukon, Northwest Territories, and Nunavut. In total, 116 cities had statistically significant higher prostate cancer incidence rates, and 265 cities had statistically significant lower incidence rates than the national average (Supplementary Tables S2 and S3). Three hundred and eighty-seven forward sortation areas (FSAs) had statistically significant higher prostate cancer incidence rates than the national average, and 609 FSAs had statistically significant lower incidence rates (Supplementary Tables S4 and S5). There were no FSAs with a population ≥5000 men that had 0 cases of prostate cancer between the years 1992 and 2010.

There were no significant correlations between prostate cancer incidence and provincial cigarette smoking rates (R2 = 0.004, *p* = 0.86), the percentage of the population that is ≥65 (R2 = 0.26, *p* = 0.14), or the percentage of the population that is obese (R2 = 0.25, *p* = 0.14) (Supplementary Table S8). On the other hand, there was a significant association between prostate cancer incidence rates by FSA and socioeconomic status (SES) quintiles. Incidence/diagnosis rates were significantly lower in the highest SES quintile compared to the lowest quintile (IRR_SES Q5 vs. Q1_ = 0.79; 95% CI 0.77–0.82) (Supplementary Table S9). In addition, surprisingly, in Canada prostate cancer diagnosis rates were significantly lower in the quintile with the highest percentage of African–Canadian/Black individuals compared to that with the lowest percentage (IRR_Black Q5 vs. Q1_ = 0.73; 95% CI 0.72–0.74) (Supplementary Table S10). Since prostate cancer diagnosis/incidence heavily depends on access to a medical system and/or likelihood of undergoing screening, we also evaluated an association between African–Canadian/Black ethnicity and prostate cancer mortality in a similar way. Our findings confirm that FSAs with the highest percentage of African–Canadian individuals had lower mortality due to prostate cancer (Supplementary Table S11).

### 3.3. Analysis of Mortality Rates and Geographic Distribution of Deaths Due to Prostate Cancer

A total of 69,655 deaths were caused by prostate cancer among Canadian men between 1992 and 2010. The overall national age-standardized mortality rate was 24.15 deaths per 100,000 men per year and the crude mortality rate was 24.13 deaths per 100,000 men per year. The mean ± SD age at the time of death was 78.4 ± 9.19. The mortality rate in men aged between 40 and 59 was 2.72 (95% CI 2.609–2.724) deaths per 100,000 men per year; 85.22 (95% CI 84.309–85.338) in men between ages 60 and 79; and 552.06 (95% CI 546.209–553.977) in men ≥ 80. There has been an overall decline in crude mortality rates by −0.19 ± 0.022 deaths per 100,000 males per year between 1992 and 2010 (*p* < 0.0001) and the age-adjusted mortality rates decreased by −0.79 ± 0.032 deaths per 100,000 males per year (*p* < 0.0001) (Figure 1). Joinpoint regression analysis of age-adjusted mortality rates revealed two significant joinpoints in 2000 and 2006, with each of the three segments showing a statistically significant decrease in annual percentage change (APC). The most significant decrease was between 2000 and 2006 with an APC of −4.9% (95% CI −5.9%–−4.0%). Temporal trend analyses for each individual province are available in Supplementary Table S12.

Seven provinces had statistically significant higher crude mortality rates than the national average: Saskatchewan, Prince Edward Island, Manitoba, Nova Scotia, New Brunswick, Newfoundland and Labrador, and British Columbia (Table 2, Figure 2B). Three provinces and two territories had statistically significant lower crude mortality rates than the national average: Ontario, Alberta, Québec, Yukon, and the Northwest Territories. In total, 28 FSAs had statistically significant higher mortality rates than the Canadian average and 1022 FSAs had statistically significant lower mortality rates (Supplementary Tables S6 and S7). There was a significant positive correlation between prostate cancer mortality rates and the percentage of the population that is ≥65 (R2 = 0.45, *p* = 0.03).

## 4. Discussion

This study allowed us to investigate the epidemiology and the geographic distribution of prostate cancer in Canada between 1992 and 2010. The overall findings from this study are consistent with those described in the Canadian/American literature, highlighting the continuity of trends between both countries [29,30]. This is the first Canadian study to identify incidence and mortality rates at the city and FSA levels across the country. We were able to identify areas of geographic clustering for this disease. The FSAs with the highest incidence rates were located within the cities with the highest incidence rates, which corroborates our results.

Previous studies have reported that prostate cancer incidences peaked in both 1993 and 2001 due to two waves of intensified screening of asymptomatic men via PSA testing, which possibly resulted in earlier diagnoses [29,31,32]. PSA testing was made available in Canada in 1986 and was widely used by the early 1990s [33,34]. It has been hypothesized that the first peak in 1993 was as a result of the introduction of the PSA screening tool, whereas the second in 2001 was partially explained by the diagnosis of Allan Rock, the former Canadian Minister of Health, with early prostate cancer as a result of the PSA screening [29]. The 95% confidence intervals for incidence rates in 1993 and 2001 do not overlap with the surrounding data points, suggesting that the peaks observed in these years could be significant. However, interestingly, our joinpoint regression analysis did not reveal any significant joinpoints. Rather, it suggests that crude incidence rates have been steadily increasing between 1992 and 2010. This increase can be explained by the widespread use of PSA screening during this time period. One study estimated that 62.77% (95% CI 59.49–66.05), 62.74% (95% CI 60.53–64.96), and 54.40% (95% CI 51.99–56.81) of men above the age of 50 in Atlantic provinces, Ontario, and Quebec, respectively, have had at least one PSA screening test in their lifetime [35]. The higher screening rates reported in Atlantic provinces and Ontario mirror the higher prostate cancer incidence rates that were observed in our study. Similarly, the lower PSA screening rates in Quebec mirror the lower incidence rates from our study. These findings support the theory that the increase in crude incidence rates is reflective of increased screening and potential overdiagnosis of men who have early-stage disease [36]. More studies are needed to further confirm this correlation and to explore its existence in other provinces. Furthermore, our results show that the age-adjusted incidence rates for prostate cancer have not significantly increased between 1992 and 2010, which suggests that the aging population also plays a role in the observed increase. The role of regional risk factors remains unclear and further analyses are required to determine whether potential harmful environmental, behavioral, and occupational exposures are involved in the pathogenesis of this malignancy.

Mortality due to prostate cancer has been significantly declining since the 1990s. In fact, crude mortality rates decreased by −0.19 ± 0.022 deaths per 100,000 males per year between 1992 and 2010. Studies have suggested that this decline is due to improved treatment modalities and earlier diagnosis [29,31,37]. However, other studies have suggested that the role of PSA screening has only a small impact on mortality, with 1 less death due to prostate cancer for every 1000 men screened over the course of 10 years [38], and that it has not been shown to significantly reduce all-cause mortality [5]. The limited impact of prostate cancer screening on mortality is supported by data from other countries, such as the United Kingdom, where there has also been a decrease in prostate cancer mortality despite having little screening [38]. The decrease in mortality rates since the early 1990s also supports this hypothesis, since it began too soon after the PSA test was first introduced [5]. Interestingly, as demonstrated by Supplementary Table S12, only certain provinces demonstrated a statistically significant decrease in mortality rates between 1992 and 2010. These provinces include Nova Scotia, Quebec, Ontario, Alberta, and British Columbia. No significant change over time was observed in the other provinces. If PSA testing was the main driver in mortality reduction, one would expect to see a more significant decrease in mortality rates in provinces with higher rates of PSA screening, such as Ontario, when compared to provinces with lower rates, such as Quebec. However, this trend was not observed. For these reasons, one might hypothesize that other factors, such as the improvement in treatment modalities, have had a more significant impact on mortality. Of note, studies have also not shown a mortality benefit from digital rectal exam screening [38].

We were also able to identify significantly lower prostate cancer incidence rates in higher SES quintiles. Similar findings have previously been reported in the literature [39]. SES is related to factors that impact the burden of prostate cancer as well as access, quality, and the use of screening and health-care services [40]. Studies also show that living in a neighborhood with a high SES is positively correlated with odds of receiving a definitive treatment for prostate cancer (OR = 1.57, 95% CI 1.01–2.42) [41]. Further, studies have found that men in lower SES groups were more likely to be diagnosed with metastatic prostate cancer and had higher mortality rates than men of higher SES (*p* < 0.05) [42].

In addition, we found that diagnosis/incidence rates were significantly lower in quintiles with higher percentage of African–Canadian/Black individuals. In contrast to this, American studies have shown that prostate adenocarcinoma occurs significantly more frequently and is deadlier in African–American men when compared to other ethnic groups [1,43]. This discrepancy could be explained by the fact that only 2.48% of the Canadian population is of African ethnicity according to the 2006 Canadian Census of Population. In addition, a significant proportion of these individuals are from the Caribbean and francophone countries (1.83% of the total Canadian population) as opposed to being of African descent, which may play an important role. Specifically, in Québec most African–Canadian individuals are francophone or bilingual immigrants from Haiti. This finding might highlight screening/diagnostic or treatment differences between Caucasian vs. African–Canadian individuals. It is also possible that traditions and behavioral patterns differ between African–American individuals in the United States vs. the African–Canadian population that impact their risk of developing/dying from the disease or factors affecting the likelihood of screening or being diagnosed with a prostate cancer.

We wish to highlight that many popular websites (https://www.cancer.ca/ or https://www.prostatecancer.ca/, accessed on 1 July 2020) or peer-reviewed studies evaluating the epidemiology of prostate cancer in Canada list African–American ethnicity as a risk factor for developing the disease. However, these claims are based on extrapolated data from the American studies [29,37,44,45]. Based on our literature search and to our knowledge, no study has explicitly compared incidence and mortality rates between ethnic groups in Canada. The absence of information on ethnicity for individual patients in Canadian cancer registries is likely the main reason for this limitation. Our results highlight the need for further Canadian studies evaluating the link between African ethnicity and the epidemiology of prostate cancer in Canada.

There are several limitations associated with large retrospective population-based studies [46]. First, it is possible that the databases had missing data or that some patients were misclassified. In addition, the databases contained only limited demographic data for each patient. They did not provide information regarding the patient’s ethnicity, occupation, smoking status, comorbid medical conditions, or fruit and vegetable consumption, which could all potentially act as confounding factors. As illustrated throughout this review, prostate cancer epidemiology studies pose methodological challenges, mostly because of the controversial use of PSA test for screening and the possibility of overdiagnosis. For this reason, further research is needed in Canada. In particular, studies should focus on high-grade and advanced-stage prostate cancer, in order to better understand the relation between mortality and PSA screening in the Canadian population. Further, study designs should consider the possible detection biases that are specific to prostate cancer, such as information on PSA screening, and be statistically adjusted accordingly. Unfortunately, the databases used did not provide any information of cancer staging or PSA screening, which is a limitation of this study.

## 5. Conclusions

In conclusion, several Canadian geographic regions of high incidence for prostate cancer have been identified through this study. These results demonstrate that external risk factors, such as environmental or lifestyle factors, are likely involved in the pathogenesis or affecting screening/diagnosis of prostate cancer in Canada.

## Figures and Tables

**Table 1:** List of prostate malignancies included in this study along with their corresponding ICD-O-3 codes, the number of cases reported in Canada between the years 1992 and 2010, and the mean age at the time of diagnosis. The same ICD-O3 code is used to represent more than one prostate cancer subtype.

ICD-O3 Code	Neoplasm	Count ^†^ (%)	Mean Age ± SD
8140	Adenocarcinoma NOS	327,195 (99.81)	68.50 ± 9.15
8201	Cribriform carcinoma NOS	90 (0.03)	70.33 ± 7.88
8260	Papillary adenocarcinoma NOS	65 (0.02)	72.92 ± 9.17
	Papillary renal cell carcinoma		
	Papillary carcinoma of thyroid		
8310	Clear cell adenocarcinoma NOS	100 (0.03)	68.36 ± 8.27
	Clear cell adenocarcinoma, mesonephroid		
8480	Mucinous adenocarcinoma	225 (0.07)	65.25 ± 10.61
	Pseudomyxoma peritonei with unknown primary site		
8490	Signet ring cell carcinoma	90 (0.03)	70.02 ± 9.51
8560	Adenosquamous carcinoma	20 (0.01)	73.55 ± 8.54
8574	Adenocarcinoma with neuroendocrine differentiation	25 (0.01)	68.15 ± 10.93
-	Total	327,810 (100)	78.4 ± 9.19

^†^ Number of cases was rounded to a multiple of 5 as per SSHRC/Statistics Canada regulations.

**Table 2:** Crude and age-adjusted incidence and mortality rates and corresponding confidence intervals for prostate cancer by province in Canada between 1992 and 2010. Incidence and mortality rates are expressed per 100,000 men per year. The 95% confidence intervals were calculated based on Poisson distributions and were adjusted for rare effects.

Province/Territory	Male Population (Rounded to 1000)	Crude Incidence Rate (95% CI)	Age-Adjusted Incidence Rate (95% CI)	Crude Mortality Rate (95% CI)	Age-Adjusted Mortality Rate (95% CI)
Alberta	1,573,000	105.78 (104.62–195.95)	118.68 (117.45–118.87)	21.08 (20.56–21.12)	23.70 (23.15–23.74)
British Columbia	2,007,000	134.18 (133.02–134.36)	118.84 (117.74–119.00)	24.61 (24.12–24.65)	20.35 (19.90–20.38)
Manitoba	575,000	120.41 (118.36–129.73)	116.73 (114.69–117.04)	30.16 (29.14–31.24)	26.13 (25.17–26.20)
New Brunswick	370,000	155.26 (152.36–155.77)	140.03 (137.24–140.50)	28.59 (27.36–28.69)	24.62 (23.46–24.71)
Newfoundland and Labrador	265,000	111.72 (108.82–112.16)	103.63 (100.78–104.04)	25.22 (23.86–25.33)	23.86 (22.51–23.97)
Nova Scotia	457,000	141.43 (138.94–141.84)	126.13 (123.74–126.51)	29.31 (28.18–29.43)	24.92 (23.87–25.01)
Ontario	5,853,000	115.85 (115.22–115.95)	111.10 (110.48–111.19)	23.19 (22.90–23.24)	21.48 (21.21–21.50)
Prince Edward Island	67,000	165.75 (158.75–167.46)	146.76 (140.15–147.94)	31.82 (28.79–32.12)	26.66 (23.88–26.92)
Quebec	3,676,000	84.56 (83.88–84.65)	77.63 (76.98–77.71)	21.04 (20.70–21.96)	19.57 (19.24–19.59)
Saskatchewan	502,000	133.10 (130.79–133.48)	123.45 (121.20–123.80)	40.78 (39.51–45.93)	31.89 (30.75–31.98)
Northwest Territories	22,000	37.08 (31.47–37.74)	63.95 (56.78–64.92)	9.57 (6.84–9.86)	21.14 (17.11–21.56)
Nunavut	15,000	8.77 (5.68–9.15)	21.38 (16.37–21.96)	-	-
Yukon	16,000	59.21 (50.88–67.36)	73.11 (63.73–74.49)	11.51 (8.02–11.92)	17.89 (13.41–18.40)

## Data Availability

The data presented in this study are available in Table 1 and Table 2 and Tables S1–S12. Additional data presented in this study are available upon request from the corresponding author.

## Supplementary Materials

The following are available online at https://www.mdpi.com/1718-7729/28/1/96/s1, Table S1: Prostate cancer incidence rates by age group for Canada as a whole between the years 1992 to 2010. Incidence rates are expressed per 100,000 men per year, Table S2: Overall prostate cancer incidence rates by city between 1992 and 2010. Incidence rates are expressed per 100,000 men per year. Only Canadian cities with statistically significant higher incidence rates compared to the national average are included in this table, Table S3: Overall prostate cancer incidence rates by city between 1992 and 2010. Incidence rates are expressed per 100,000 men per year. Only Canadian cities with statistically significant lower incidence rates compared to the national average are included in this table, Table S4: Overall prostate cancer incidence rates by Forward Sortation Area (FSA) between 1992 and 2010. Incidence rates are expressed per 100,000 men per year. Only FSAs with statistically significant higher incidence rates compared to the national average are included in this table, Table S5: Overall prostate cancer incidence rates by Forward Sortation Area (FSA) between 1992 and 2010. Incidence rates are expressed per 100,000 men per year. Only FSAs with statistically significant lower incidence rates compared to the national average are included in this table, Table S6: Overall prostate cancer mortality rates by Forward Sortation Area (FSA) between 1992 and 2010. Mortality rates are expressed per 100,000 men per year. Only FSAs with statistically significant higher mortality rates compared to the national average are included in this table. Table S7: Overall prostate cancer mortality rates by Forward Sortation Area (FSA) between 1992 and 2010. Mortality rates are expressed per 100,000 men per year. Only FSAs with statistically significant lower mortality rates compared to the national average are included in this table, Table S8: Percentage of each Canadian province that is obese, that is above the age of 65, and that is of Black ethnicity, Table S9: Incidence rate ratio (IRR) analyses for each FSA socioeconomic status quintile. The IRRs compare the given quintile to Quintile 1 (lowest socioeconomic status). An IRR >1 for a given quintile represents an incidence rate that is higher than that of Quintile 1. An IRR <1 for a given quintile represents an incidence rate that is lower than that of Quintile 1, Table S10: Incidence rate ratio (IRR) analyses for each FSA Black visible minority quintile. The IRRs compare the given quintile to Quintile 1 (lowest percentage of Black individuals). An IRR >1 for a given quintile represents an incidence rate that is higher than that of Quintile 1. An IRR <1 for a given quintile represents an incidence rate that is lower than that of Quintile 1, Table S11: Analyses of ratios of mortality rates for each FSA Black visible minority quintile. The ratios compare the given quintile to Quintile 1 (lowest percentage of Black individuals). A ratio >1 for a given quintile represents a mortality rate that is higher than that of Quintile 1. A ratio <1 for a given quintile represents a mortality rate that is lower than that of Quintile 1, Table S12: Provincial mortality rates (per 100,000 men per year) of all deaths by prostate cancer between 1992 and 2010 with linear regression analysis of the mortality rate over time. The slope of the linear regression analysis represents the overall yearly change in mortality rates and is expressed as deaths per 100,000 men per year.
