# Supplementary material for: Incidence and Mortality of Prostate Cancer in Canada during 1992–2010
Source: Curr Oncol. 2021 Feb 21;28(1):978–90. doi: 10.3390/curroncol28010096 (PMC7985768; doi:10.3390/curroncol28010096)
Supplement: Supplementary file 1 [file curroncol-28-00096-s001.pdf]

Supplementary Table 1. Prostate cancer incidence rates by age group for Canada for years 1992 to 2010. Incidence rates are expressed per 100,000 men per year.

Age group	Count †	Male Population	Incidence per 100,000 men (95% CI)
0-19	5	4,062,000	0.006 (0.002-0.007)
20-39	70	4,631,000	0.08 (0.06-0.08)
40-59	55,165	4,304,000	67.46 (66.90-67.52)
60-79	233,685	2,064,000	595.89 (593.48-596.71)
≥80	38,845	324,000	631.01 (624.75-633.20)

† Number of cases was rounded to a multiple of 5 as per SSHRC/Statistics Canada regulations.

Supplementary Table 2. Overall prostate cancer incidence rates by city between 1992 and 2010. Incidence rates are expressed per 100,000 men per year. Only Canadian cities with statistically significant higher incidence rates compared to the national average are included in this table.

City	Cases †	Male Population (rounded to 1,000)	Incidence Rate (95% CI)
NORTH SAANICH	325	5,000	342.11 (305.92-381.40)
SIDNEY	300	5,000	315.79 (281.06-353.62)
BATHURST	355	6,000	311.40 (279.85-345.54)
COMOX	285	5,000	300.00 (266.18-336.93)
WHITE ROCK	445	8,000	292.76 (266.19-321.27)
OAK BAY	425	8,000	279.61 (253.65-307.49)
WEST VANCOUVER	965	19,000	267.31 (250.71-284.73)
POWELL RIVER	295	6,000	258.77 (230.08-290.05)
CENTRAL SAANICH	335	7,000	251.88 (225.63-280.35)
YARMOUTH	225	5,000	236.84 (206.90-269.89)
SUMMERLAND	225	5,000	236.84 (206.90-269.89)
COURTENAY	400	9,000	233.92 (211.55-258.00)
KINCARDINE	220	5,000	231.58 (201.99-264.29)
PARKSVILLE	220	5,000	231.58 (201.99-264.29)
NIAGARA-ON-THE-LAKE	305	7,000	229.32 (204.31-256.56)
YORKTON	305	7,000	229.32 (204.31-256.56)

PENTICTON	640	15,000	224.56 (207.50-242.65)
WASAGA BEACH	285	7,000	214.29 (190.13-240.66)
CHESTER	200	5,000	210.53 (182.36-241.81)
MEAFORD	200	5,000	210.53 (182.36-241.81)
PORT COLBORNE	355	9,000	207.60 (186.57-230.36)
SWIFT CURRENT	275	7,000	206.77 (183.05-232.71)
HIGH RIVER	195	5,000	205.26 (177.46-236.18)
TILLSONBURG	270	7,000	203.01 (179.51-228.72)
NORTH VANCOUVER	1,510	40,000	198.68 (188.79-208.96)
COLLINGWOOD	300	8,000	197.37 (175.66-221.01)
FORT ERIE	520	14,000	195.49 (179.05-213.04)
SALMON ARM	260	7,000	195.49 (172.45-220.75)
ELLIOT LAKE	220	6,000	192.98 (168.32-220.24)
BROCKVILLE	365	10,000	192.11 (172.90-212.86)
PELHAM	290	8,000	190.79 (169.46-214.06)
LUNENBURG	470	13,000	190.28 (173.47-208.29)
CAMROSE	250	7,000	187.97 (165.39-212.77)
OWEN SOUND	350	10,000	184.21 (165.42-204.56)
EDMUNDSTON	280	8,000	184.21 (163.26-207.10)
TRURO	175	5,000	184.21 (157.93-213.62)
MOOSE JAW	550	16,000	180.92 (166.11-196.69)
VERNON	540	16,000	177.63 (162.96-193.27)
SAINT JOHN	1,100	33,000	175.44 (165.22-186.12)

PICTOU, SUBD. C	165	5,000	173.68 (148.19-202.30)
LANGLEY	1,475	45,000	172.51 (163.82-181.55)
WELLAND	785	24,000	172.15 (160.32-184.62)
COBOURG	260	8,000	171.05 (150.89-193.16)
SARNIA	1,130	35,000	169.92 (160.16-180.13)
CAMPBELL RIVER	445	14,000	167.29 (152.11-183.58)
SAANICH	1,600	51,000	165.12 (157.13-173.41)
MONCTON	940	30,000	164.91 (154.54-175.80)
CORNER BROOK	310	10,000	163.16 (145.50-182.37)
NIAGARA FALLS	1,205	39,000	162.62 (153.56-172.07)
PORTAGE LA PRAIRIE	185	6,000	162.28 (139.74-187.43)
THUNDER BAY	1,615	53,000	160.38 (152.65-168.39)
NORTH COWICHAN	390	13,000	157.89 (142.61-174.37)
MIRAMICHI	270	9,000	157.89 (139.62-177.90)
LAMBTON SHORES	150	5,000	157.89 (133.64-185.28)
LAC STE. ANNE COUNTY	150	5,000	157.89 (133.64-185.28)
KELOWNA	1,455	49,000	156.28 (148.36-164.53)
VICTORIA	1,030	35,000	154.89 (145.57-164.64)
ESQUIMALT	235	8,000	154.61 (135.47-175.69)
CAPITAL, SUBD. A	175	6,000	153.51 (131.61-178.01)
BRIGHTON	145	5,000	152.63 (128.80-179.59)
CHILLIWACK	955	33,000	152.31 (142.80-162.29)

WOODSTOCK	485	17,000	150.15 (137.09-164.13)
ROTHESAY	170	6,000	149.12 (127.55-173.30)
BROCK	170	6,000	149.12 (127.55-173.30)
PEMBROKE	170	6,000	149.12 (127.55-173.30)
MOUNTAIN VIEW COUNTY	170	6,000	149.12 (127.55-173.30)
FREDERICTON	675	24,000	148.03 (137.07-159.63)
NEW TECUMSETH	365	13,000	147.77 (133.00-163.74)
CORNWALL	615	22,000	147.13 (135.73-159.23)
SUMMERSIDE	195	7,000	146.62 (126.76-168.70)
LEDUC COUNTY	195	7,000	146.62 (126.76-168.70)
CHARLOTTETOWN	415	15,000	145.61 (131.94-160.32)
WILMOT	220	8,000	144.74 (126.24-165.18)
WETASKIWIN COUNTY NO. 10	165	6,000	144.74 (123.49-168.58)
NANAIMO	1,015	37,000	144.38 (135.63-153.54)
ROCKY VIEW NO. 44	435	16,000	143.09 (129.96-157.19)
WEST HANTS	190	7,000	142.86 (123.27-164.68)
BRACEBRIDGE	190	7,000	142.86 (123.27-164.68)
NORTH BAY	705	26,000	142.71 (132.37-153.65)
QUEENS	135	5,000	142.11 (119.15-168.20)
WEYBURN	135	5,000	142.11 (119.15-168.20)

YELLOWHEAD COUNTY	135	5,000	142.11 (119.15-168.20)
GRIMSBY	295	11,000	141.15 (125.50-158.21)
SAULT STE. MARIE	990	37,000	140.83 (132.19-149.88)
GRANBY	560	21,000	140.35 (128.97-152.47)
WETASKIWIN	160	6,000	140.35 (119.45-163.86)
BRANTFORD	1,145	43,000	140.15 (132.15-148.51)
ST. CATHARINES	1,665	63,000	139.10 (132.50-145.94)
DIEPPE	210	8,000	138.16 (120.10-158.16)
DELTA	1,255	48,000	137.61 (130.10-145.44)
LETHBRIDGE	915	35,000	137.59 (128.82-146.81)
STRATFORD	365	14,000	137.22 (123.50-152.04)
BRANDON	520	20,000	136.84 (125.33-149.13)
LINCOLN	260	10,000	136.84 (120.71-154.53)
COLDSTREAM	130	5,000	136.84 (114.33-162.49)
ST. THOMAS	440	17,000	136.22 (123.79-149.57)
BURLINGTON	1,965	76,000	136.08 (130.13-142.23)
LACHUTE	155	6,000	135.96 (115.40-159.13)
RIVERVIEW	205	8,000	134.87 (117.04-154.65)
UXBRIDGE	230	9,000	134.50 (117.68-153.06)
THOROLD	230	9,000	134.50 (117.68-153.06)
HUNTSVILLE	230	9,000	134.50 (117.68-153.06)
FOOTHILLS NO. 31	230	9,000	134.50 (117.68-153.06)

PORT ALBERNI	230	9,000	134.50 (117.68-153.06)
MEDICINE HAT	660	26,000	133.60 (123.60-144.20)
LONDON	4,200	166,000	133.16 (129.17-137.25)
CAPE BRETON	1,255	50,000	132.11 (124.90-139.62)
WINDSOR	2,530	101,000	131.84 (126.75-137.08)
HALDIMAND	550	22,000	131.58 (120.81-143.05)
KINGS, SUBD. A	275	11,000	131.58 (116.49-148.09)
SCUGOG	250	10,000	131.58 (115.77-148.94)
COLWOOD	175	7,000	131.58 (112.81-152.58)
WHITCHURCH-STOUFFVILLE	320	13,000	129.55 (115.75-144.56)
REGINA	2,165	88,000	129.49 (124.09-135.06)
SAINT-LAMBERT	245	10,000	128.95 (113.31-146.15)
ESSEX	245	10,000	128.95 (113.31-146.15)
WATERLOO	1,070	44,000	127.99 (120.44-135.90)
KAMLOOPS	935	39,000	126.18 (118.22-134.54)
NEW WESTMINSTER	670	28,000	125.94 (116.58-135.85)
PETERBOROUGH	810	34,000	125.39 (116.90-134.33)
BURNABY	2,305	98,000	123.79 (118.79-128.95)
WINNIPEG	7,190	306,000	123.67 (120.82-126.56)
OSHAWA	1,575	69,000	120.14 (114.28-126.22)

† Number of cases was rounded to a multiple of 5 as per SSHRC/Statistics Canada regulations.

Supplementary Table 3. Overall prostate cancer incidence rates by city between 1992 and 2010. Incidence rates are expressed per 100,000 men per year. Only Canadian cities with statistically significant lower incidence rates compared to the national average are included in this table.

City	Cases †	Male Population (rounded to 1,000)	Incidence Rate (95% CI)
COQUITLAM	1,110	56,000	104.32 (98.28-110.65)
EDMONTON	6,680	349,000	100.74 (98.34-103.18)
OAKVILLE	1,445	76,000	100.07 (94.98-105.37)
VANCOUVER	5,215	275,000	99.81 (97.12-102.55)
AURORA	395	21,000	99.00 (89.47-109.26)
BELLEVILLE	430	23,000	98.40 (89.32-108.15)
HALIFAX	3,355	180,000	98.10 (94.81-101.48)
CALGARY	8,630	465,000	97.68 (95.63-99.76)
PORT MOODY	240	13,000	97.17 (85.26-110.27)
KINGSTON	1,045	57,000	96.49 (90.73-102.52)
ORANGEVILLE	220	12,000	96.49 (84.16-110.12)
CHATHAM-KENT	950	52,000	96.15 (90.14-102.47)
NEWMARKET	620	34,000	95.98 (88.57-103.84)
POINTE-CLAIRE	255	14,000	95.86 (84.46-108.38)
CONCEPTION BAY SOUTH	200	11,000	95.69 (82.89-109.91)

BARRIE	970	54,000	94.54 (88.69-100.68)
PICKERING	750	42,000	93.98 (87.38-100.96)
WHITBY	855	48,000	93.75 (87.57-100.25)
GRANDE PRAIRIE COUNTY NO. 1	160	9,000	93.57 (79.63-109.24)
Boucherville	335	19,000	92.80 (83.13-103.29)
RICHMOND	1,445	82,000	92.75 (88.03-97.66)
PRINCE GEORGE	630	36,000	92.11 (85.05-99.59)
L'ANCIENNE LORETTE	140	8,000	92.11 (77.48-108.69)
MISSISSIPPI MILLS	105	6,000	92.11 (75.33-111.50)
MAPLE RIDGE	575	33,000	91.71 (84.36-99.52)
KAWARTHA LAKES	625	36,000	91.37 (84.35-98.83)
TROIS-RIVIERES	380	22,000	90.91 (82.00-100.53)
RIVIERE-DU-LOUP	155	9,000	90.64 (76.94-106.09)
SPRUCE GROVE	155	9,000	90.64 (76.94-106.09)
GRAND-MERE	120	7,000	90.23 (74.81-107.89)
BRADFORD WEST GWILLIMBURY	205	12,000	89.91 (78.02-103.10)
SASKATOON	1,685	99,000	89.58 (85.35-93.96)
VANIER	85	5,000	89.47 (71.47-110.64)
SAINT-SAUVEUR	85	5,000	89.47 (71.47-110.64)

SOUTH DUNDAS	85	5,000	89.47 (71.47-110.64)
MALAHIDE	85	5,000	89.47 (71.47-110.64)
DAWSON CREEK	85	5,000	89.47 (71.47-110.64)
MIDDLESEX CENTRE	135	8,000	88.82 (74.47-105.12)
CHATEAUGUAY	350	21,000	87.72 (78.77-97.41)
TRENT HILLS	100	6,000	87.72 (71.37-106.69)
MARKHAM	1,910	116,000	86.66 (82.82-90.64)
SIMCOE	115	7,000	86.47 (71.39-103.79)
PRINCE RUPERT	115	7,000	86.47 (71.39-103.79)
CAMBRIDGE	910	56,000	85.53 (80.06-91.27)
MOUNT PEARL	195	12,000	85.53 (73.94-98.41)
OKOTOKS	130	8,000	85.53 (71.46-101.56)
SQUAMISH	130	8,000	85.53 (71.46-101.56)
AJAX	665	41,000	85.37 (79.00-92.11)
OTTAWA	6,455	400,000	84.93 (82.87-87.03)
SAINTE-AGATHE-DES-MONTS	80	5,000	84.21 (66.77-104.81)
ELIZABETHTOWN-KITLEY	80	5,000	84.21 (66.77-104.81)
GREY HIGHLANDS	80	5,000	84.21 (66.77-104.81)
CAP-DE-LA-MADELEINE	255	16,000	83.88 (73.90-94.83)

LANGFORD	175	11,000	83.73 (71.79-97.10)
LAVAL	2,790	176,000	83.43 (80.37-86.59)
SOUTH STORMONT	95	6,000	83.33 (67.42-101.87)
RED DEER	580	37,000	82.50 (75.92-89.50)
ALMA	235	15,000	82.46 (72.25-93.70)
SAINT-JEROME	500	32,000	82.24 (75.19-89.77)
DEUX-MONTAGNES	125	8,000	82.24 (68.45-97.98)
CENTRAL OKANAGAN J	125	8,000	82.24 (68.45-97.98)
MILTON	390	25,000	82.11 (74.16-90.67)
MISSISSAUGA	4,830	312,000	81.48 (79.20-83.81)
SAINTE-FOY	525	34,000	81.27 (74.47-88.53)
LA PRAIRIE	150	10,000	78.95 (66.82-92.64)
SUNSHINE COAST, SUBD. A	105	7,000	78.95 (64.57-95.57)
SAINTE-MARIE	90	6,000	78.95 (63.48-97.04)
GUELPH/ERAMOSA	90	6,000	78.95 (63.48-97.04)
WELLINGTON NORTH	90	6,000	78.95 (63.48-97.04)
SAINT-ROMUALD	75	5,000	78.95 (62.10-98.96)
SAINT-ANTOINE	75	5,000	78.95 (62.10-98.96)
ROBERVAL	75	5,000	78.95 (62.10-98.96)

SOOKE	75	5,000	78.95 (62.10-98.96)
BRAMPTON	2,820	192,000	77.30 (74.48-80.21)
PORT COQUITLAM	375	26,000	75.91 (68.42-84.00)
LASALLE	490	34,000	75.85 (69.28-82.87)
SHAWINIGAN	345	24,000	75.66 (67.88-84.08)
LAKESHORE	230	16,000	75.66 (66.20-86.09)
LINDSAY	115	8,000	75.66 (62.46-90.82)
CENTRAL KOOTENAY, SUBD. B	115	8,000	75.66 (62.46-90.82)
MATANE	100	7,000	75.19 (61.18-91.45)
NORTH GRENVILLE	100	7,000	75.19 (61.18-91.45)
AMOS	85	6,000	74.56 (59.56-92.20)
PERTH EAST	85	6,000	74.56 (59.56-92.20)
CAPITAL, SUBD. C	85	6,000	74.56 (59.56-92.20)
GRANDE PRAIRIE	310	22,000	74.16 (66.14-82.90)
MIRABEL	225	16,000	74.01 (64.66-84.34)
SAINT-COLOMBAN	70	5,000	73.68 (57.44-93.10)
BROCKTON	70	5,000	73.68 (57.44-93.10)
HINTON	70	5,000	73.68 (57.44-93.10)
SAINT-JEAN-SUR-RICHELIEU	615	44,000	73.56 (67.86-79.62)

TORONTO	16,935	1,219,000	73.12 (72.02-74.23)
CENTRE WELLINGTON	180	13,000	72.87 (62.62-84.33)
PRINCE EDWARD	165	12,000	72.37 (61.75-84.29)
GREENFIELD PARK	110	8,000	72.37 (59.48-87.22)
LACHINE	260	19,000	72.02 (63.53-81.33)
CHARLESBOURG	465	34,000	71.98 (65.59-78.83)
SAINT-LAURENT	490	36,000	71.64 (65.43-78.27)
VERDUN	380	28,000	71.43 (64.43-78.98)
ROSEMERE	95	7,000	71.43 (57.79-87.32)
NANAIMO, SUBD. A	95	7,000	71.43 (57.79-87.32)
DOLLARD-DES-ORMEAUX	325	24,000	71.27 (63.73-79.46)
VAUGHAN	1,400	104,000	70.85 (67.19-74.66)
SAINT-GEORGES	200	15,000	70.18 (60.79-80.60)
ESSA	120	9,000	70.18 (58.18-83.91)
TRACY	80	6,000	70.18 (55.64-87.34)
SAINTE-SOPHIE	80	6,000	70.18 (55.64-87.34)
TERRACE	80	6,000	70.18 (55.64-87.34)
MONTREAL-NORD	505	38,000	69.94 (63.98-76.32)
SAINT-EUSTACHE	265	20,000	69.74 (61.59-78.66)
BROSSARD	490	37,000	69.70 (63.67-76.15)

TROIS-RIVIERES-OUEST	145	11,000	69.38 (58.55-81.63)
CHAMBLY	145	11,000	69.38 (58.55-81.63)
CENTRAL OKANAGAN, SUBD. B	145	11,000	69.38 (58.55-81.63)
RICHMOND HILL	935	71,000	69.31 (64.94-73.90)
GREATER SUDBURY	1,005	77,000	68.69 (64.51-73.08)
MERCIER	65	5,000	68.42 (52.81-87.21)
LAFONTAINE	65	5,000	68.42 (52.81-87.21)
NORTH DUNDAS	65	5,000	68.42 (52.81-87.21)
SMITH	65	5,000	68.42 (52.81-87.21)
MOORE	65	5,000	68.42 (52.81-87.21)
BAIE-COMEAU	155	12,000	67.98 (57.70-79.57)
LORETTEVILLE	90	7,000	67.67 (54.41-83.18)
SAINTE-ANNE-DES-PLAINES	90	7,000	67.67 (54.41-83.18)
SALABERRY-DE-VALLEYFIELD	240	19,000	66.48 (58.34-75.45)
KIRKLAND	125	10,000	65.79 (54.76-78.39)
DUNNVILLE	75	6,000	65.79 (51.75-82.47)
BROOKS	75	6,000	65.79 (51.75-82.47)

SOREL-TRACY	210	17,000	65.02 (56.52-74.43)
CHICOUTIMI	370	30,000	64.91 (58.47-71.88)
SAINT-LEONARD	405	33,000	64.59 (58.45-71.20)
LONGUEUIL	1,360	111,000	64.49 (61.10-68.01)
BEDFORD	85	7,000	63.91 (51.05-79.03)
GASPE	85	7,000	63.91 (51.05-79.03)
CANDIAC	85	7,000	63.91 (51.05-79.03)
NANTICOKE	145	12,000	63.60 (53.67-74.83)
ROUYN-NORANDA	240	20,000	63.16 (55.42-71.67)
STRATHROY-CARADOC	120	10,000	63.16 (52.36-75.52)
CHARNY	60	5,000	63.16 (48.20-81.30)
SAINTE-MARTHE-SUR-LE-LAC	60	5,000	63.16 (48.20-81.30)
HURON EAST	60	5,000	63.16 (48.20-81.30)
TACHE	60	5,000	63.16 (48.20-81.30)
ANJOU	215	18,000	62.87 (54.74-71.85)
DELHI	95	8,000	62.50 (50.57-76.40)
RIMOUSKI	245	21,000	61.40 (53.95-69.59)
QUINTE WEST	245	21,000	61.40 (53.95-69.59)
L'ASSOMPTION	105	9,000	61.40 (50.22-74.33)
LES ILES-DE-LA-MADELEINE	70	6,000	61.40 (47.87-77.58)

BEAUHARNOIS	70	6,000	61.40 (47.87-77.58)
DRUMMONDVILLE	395	34,000	61.15 (55.26-67.48)
MASCOUCHE	195	17,000	60.37 (52.19-69.47)
REPENTIGNY	435	38,000	60.25 (54.72-66.19)
SHERBROOKE	835	73,000	60.20 (56.19-64.43)
CHATHAM	240	21,000	60.15 (52.78-68.26)
SAINTE-JULIE	160	14,000	60.15 (51.19-70.23)
AIRDRIE	160	14,000	60.15 (51.19-70.23)
CAP-ROUGE	80	7,000	60.15 (47.70-74.86)
GREATER NAPANEE	80	7,000	60.15 (47.70-74.86)
DUNDAS	125	11,000	59.81 (49.78-71.26)
JONQUIERE	305	27,000	59.45 (52.97-66.52)
SAINTE-CATHERINE	90	8,000	59.21 (47.61-72.78)
SOUTH FRONTENAC	100	9,000	58.48 (47.58-71.13)
FORT ST. JOHN	100	9,000	58.48 (47.58-71.13)
OROMOCTO	55	5,000	57.89 (43.61-75.36)
LORRAINE	55	5,000	57.89 (43.61-75.36)
SAINT-FELICIEN	55	5,000	57.89 (43.61-75.36)
GLANBROOK	55	5,000	57.89 (43.61-75.36)

LAURENTIAN VALLEY	55	5,000	57.89 (43.61-75.36)
SAINT-CONSTANT	130	12,000	57.02 (47.64-67.70)
THE NATION	65	6,000	57.02 (44.00-72.67)
RIDEAU	65	6,000	57.02 (44.00-72.67)
NORTH OKANAGAN, SUBD. B	65	6,000	57.02 (44.00-72.67)
ETOBICOKE	1,710	158,000	56.96 (54.29-59.73)
VARENNES	105	10,000	55.26 (45.20-66.90)
OUTREMONT	105	10,000	55.26 (45.20-66.90)
LA BAIE	105	10,000	55.26 (45.20-66.90)
DARTMOUTH	325	31,000	55.18 (49.34-61.52)
SOREL	115	11,000	55.02 (45.43-66.05)
CLARENCE-ROCKLAND	115	11,000	55.02 (45.43-66.05)
SEPT-ILES	125	12,000	54.82 (45.64-65.32)
BOISBRIAND	135	13,000	54.66 (45.83-64.69)
CARIBOO, SUBD. B	135	13,000	54.66 (45.83-64.69)
MONTREAL	8,185	790,000	54.53 (53.36-55.72)
VAL-D'OR	165	16,000	54.28 (46.31-63.22)
BEAUPORT	360	35,000	54.14 (48.69-60.03)
NORTH YORK	2,880	281,000	53.94 (51.99-55.95)

BLAINVILLE	215	21,000	53.88 (46.92-61.59)
QUEBEC	2,460	241,000	53.72 (51.62-55.89)
SAINT-LUC	100	10,000	52.63 (42.82-64.01)
VANIER	80	8,000	52.63 (41.73-65.50)
LOYALIST	80	8,000	52.63 (41.73-65.50)
SAINT-BASILE-LE-GRAND	70	7,000	52.63 (41.03-66.50)
MONT-LAURIER	70	7,000	52.63 (41.03-66.50)
COWANSVILLE	60	6,000	52.63 (40.16-67.75)
PINCOURT	60	6,000	52.63 (40.16-67.75)
WALLACEBURG	60	6,000	52.63 (40.16-67.75)
L'ILE-PERROT	50	5,000	52.63 (39.06-69.39)
MERSEA	50	5,000	52.63 (39.06-69.39)
PEACE RIVER, SUBD. C	50	5,000	52.63 (39.06-69.39)
NEPEAN	545	56,000	51.22 (47.01-55.71)
ANCASTER	115	12,000	50.44 (41.64-60.54)
SAINT-AUGUSTIN-DE-DESMAURES	85	9,000	49.71 (39.70-61.46)
SAINT-NICOLAS	75	8,000	49.34 (38.81-61.85)
OSGOODE	75	8,000	49.34 (38.81-61.85)
WEST CARLETON	75	8,000	49.34 (38.81-61.85)
PETAWAWA	75	8,000	49.34 (38.81-61.85)

BELLEFEUILLE	65	7,000	48.87 (37.72-62.29)
THOMPSON	65	7,000	48.87 (37.72-62.29)
PIERREFONDS	240	26,000	48.58 (42.63-55.13)
VAUDREUIL-DORION	110	12,000	48.25 (39.65-58.15)
HALIFAX, SUBD. A	55	6,000	48.25 (36.35-62.80)
HANOVER	55	6,000	48.25 (36.35-62.80)
EAST YORK	450	50,000	47.37 (43.09-51.95)
SAINT-LIN	45	5,000	47.37 (34.55-63.38)
NOTRE-DAME-DE-L'ILE-PERROT	45	5,000	47.37 (34.55-63.38)
MAPLETON	45	5,000	47.37 (34.55-63.38)
WHISTLER	45	5,000	47.37 (34.55-63.38)
CARIBOO, SUBD. A	80	9,000	46.78 (37.10-58.23)
LEVIS	575	65,000	46.56 (42.83-50.52)
SEPT-ILES	115	13,000	46.56 (38.44-55.89)
SCARBOROUGH	2,310	269,000	45.20 (43.37-47.08)
SAINT-LIN - LAURENTIDES	60	7,000	45.11 (34.43-58.07)
HULL	265	31,000	44.99 (39.74-50.75)
SAINT-HUBERT	320	38,000	44.32 (39.60-49.45)
STONEY CREEK	225	27,000	43.86 (38.32-49.98)
LAVALTRIE	50	6,000	43.86 (32.55-57.82)

STRATHROY	50	6,000	43.86 (32.55-57.82)
TRENTON	65	8,000	42.76 (33.00-54.51)
CENTRAL OKANAGAN H	65	8,000	42.76 (33.00-54.51)
YORK	555	69,000	42.33 (38.88-46.01)
HALIFAX, SUBD. E	80	10,000	42.11 (33.39-52.40)
WHITECOURT	40	5,000	42.11 (30.08-57.34)
MACKENZIE NO. 23	40	5,000	42.11 (30.08-57.34)
TERREBONNE	395	50,000	41.58 (37.58-45.89)
DOLBEAU-MISTASSINI	55	7,000	41.35 (31.15-53.83)
FLAMBOROUGH	130	17,000	40.25 (33.63-47.79)
LACHENAIE	75	10,000	39.47 (31.05-49.48)
GOULBOURN	75	10,000	39.47 (31.05-49.48)
WHITEHORSE	75	10,000	39.47 (31.05-49.48)
HALIFAX, SUBD. B	60	8,000	39.47 (30.12-50.81)
SIDNEY	60	8,000	39.47 (30.12-50.81)
PARADISE	45	6,000	39.47 (28.79-52.82)
BUCKINGHAM	45	6,000	39.47 (28.79-52.82)
COLD LAKE	45	6,000	39.47 (28.79-52.82)
SUDBURY	320	44,000	38.28 (34.20-42.71)
ROCK FOREST	65	9,000	38.01 (29.34-48.45)
YELLOWKNIFE	65	9,000	38.01 (29.34-48.45)

FRASER-FORT GEORGE, SUBD. A	50	7,000	37.59 (27.90-49.56)
SAINT-EMILE	35	5,000	36.84 (25.66-51.24)
WALDEN	35	5,000	36.84 (25.66-51.24)
TEMISKAMING SHORES	35	5,000	36.84 (25.66-51.24)
GREATER VANCOUVER A	35	5,000	36.84 (25.66-51.24)
CENTRAL OKANAGAN G	35	5,000	36.84 (25.66-51.24)
GLOUCESTER	355	51,000	36.64 (32.92-40.65)
FLEURIMONT	55	8,000	36.18 (27.26-47.10)
SAINT-LAZARE	55	8,000	36.18 (27.26-47.10)
L'ILE-BIZARD	45	7,000	33.83 (24.68-45.27)
PITTSBURGH	45	7,000	33.83 (24.68-45.27)
HALIFAX, SUBD. C	165	26,000	33.40 (28.50-38.90)
LA PLAINE	50	8,000	32.89 (24.42-43.37)
AYLMER	105	17,000	32.51 (26.59-39.35)
VAL-BELAIR	60	10,000	31.58 (24.10-40.65)
VAL-DES-MONTS	30	5,000	31.58 (21.31-45.08)
PEACE RIVER, SUBD. B	30	5,000	31.58 (21.31-45.08)
SAGUENAY	420	71,000	31.13 (28.23-34.26)

MAIDSTONE	35	6,000	30.70 (21.38-42.70)
WOOD BUFFALO	150	26,000	30.36 (25.70-35.63)
HALIFAX, SUBD. D	115	20,000	30.26 (24.99-36.33)
LE GARDEUR	50	9,000	29.24 (21.70-38.55)
KANATA	125	24,000	27.41 (22.82-32.66)
TROIS-RIVIERES	315	62,000	26.74 (23.87-29.86)
CUMBERLAND	120	24,000	26.32 (21.82-31.47)
SAINT-JEAN-CHRYSOSTOME	40	8,000	26.32 (18.80-35.83)
KENORA	40	8,000	26.32 (18.80-35.83)
CLARENCE	25	5,000	26.32 (17.03-38.85)
GATINEAU	550	123,000	23.53 (21.61-25.59)
RAYSIDE-BALFOUR	35	8,000	23.03 (16.04-32.02)
ERNESTOWN	30	7,000	22.56 (15.22-32.20)
NICKEL CENTRE	30	7,000	22.56 (15.22-32.20)
SAINT-NICEPHORE	20	5,000	21.05 (12.86-32.51)
VALLEY EAST	35	12,000	15.35 (10.69-21.35)

† Number of cases was rounded to a multiple of 5 as per SSHRC/Statistics Canada regulations.

Supplementary Table 4. Overall prostate cancer incidence rates by Forward Sortation Area (FSA) between 1992 and 2010. Incidence rates are expressed per 100,000 men per year. Only FSAs with statistically significant higher incidence rates compared to the national average are included in this table.

FSA	Cases †	Male Population (rounded to 1,000)	Incidence Rate (95% CI)
V8L	640	10,000	336.84 (311.25-363.98)
V7V	425	7,000	319.55 (289.89-351.42)
V4B	470	8,000	309.21 (281.88-338.47)
V4A	795	16,000	261.51 (243.65-280.34)
V9K	345	7,000	259.40 (232.75-288.27)
V9N	630	13,000	255.06 (235.53-275.78)
V0X	480	10,000	252.63 (230.53-276.28)
V0S	235	5,000	247.37 (216.75-281.10)
V0H	1,155	25,000	243.16 (229.34-257.60)
S3N	360	8,000	236.84 (213.01-262.62)
V1R	225	5,000	236.84 (206.90-269.89)
V8A	400	9,000	233.92 (211.55-258.00)
N0H	975	22,000	233.25 (218.84-248.37)
V9M	310	7,000	233.08 (207.86-260.53)
V8S	350	8,000	230.26 (206.77-255.70)
E1N	215	5,000	226.32 (197.07-258.67)
S0H	515	12,000	225.88 (206.79-246.25)

V9P	425	10,000	223.68 (202.92-246.00)
N7V	255	6,000	223.68 (197.07-252.89)
V2A	715	17,000	221.36 (205.43-238.20)
N2Z	210	5,000	221.05 (192.16-253.06)
K1H	290	7,000	218.05 (193.67-244.64)
S0N	450	11,000	215.31 (195.87-236.15)
E2A	365	9,000	213.45 (192.11-236.51)
S0A	1,135	28,000	213.35 (201.11-226.13)
V8V	405	10,000	213.16 (192.90-234.96)
L3K	360	9,000	210.53 (189.34-233.44)
S9H	320	8,000	210.53 (188.09-234.90)
V8P	320	8,000	210.53 (188.09-234.90)
L7N	240	6,000	210.53 (184.73-238.91)
R0J	515	13,000	208.50 (190.88-227.31)
E2M	355	9,000	207.60 (186.57-230.36)
B0S	355	9,000	207.60 (186.57-230.36)
L0S	945	24,000	207.24 (194.23-220.88)
N7A	235	6,000	206.14 (180.62-234.25)
V4P	235	6,000	206.14 (180.62-234.25)
S6H	540	14,000	203.01 (186.24-220.87)
V4M	270	7,000	203.01 (179.51-228.72)
B2H	270	7,000	203.01 (179.51-228.72)
M3B	230	6,000	201.75 (176.52-229.58)

K0M	880	23,000	201.37 (188.29-215.13)
P0A	305	8,000	200.66 (178.77-224.49)
L2T	190	5,000	200.00 (172.57-230.55)
V8Y	190	5,000	200.00 (172.57-230.55)
V8K	190	5,000	200.00 (172.57-230.55)
M4N	265	7,000	199.25 (175.98-224.74)
V8M	260	7,000	195.49 (172.45-220.75)
L2E	370	10,000	194.74 (175.40-215.63)
K6V	480	13,000	194.33 (177.33-212.52)
V8N	440	12,000	192.98 (175.37-211.89)
N2J	330	9,000	192.98 (172.72-214.97)
P5A	220	6,000	192.98 (168.32-220.24)
R3K	220	6,000	192.98 (168.32-220.24)
S4S	585	16,000	192.43 (177.15-208.68)
B1P	255	7,000	191.73 (168.92-216.76)
T2L	255	7,000	191.73 (168.92-216.76)
N6H	540	15,000	189.47 (173.83-206.15)
N6A	180	5,000	189.47 (162.80-219.27)
V7S	180	5,000	189.47 (162.80-219.27)
N3Y	395	11,000	189.00 (170.81-208.58)
E3V	215	6,000	188.60 (164.23-215.56)
M4W	215	6,000	188.60 (164.23-215.56)
S0G	1,145	32,000	188.32 (177.57-199.55)

V0R	1,145	32,000	188.32 (177.57-199.55)
M4V	250	7,000	187.97 (165.39-212.77)
V1Y	535	15,000	187.72 (172.15-204.32)
L9Y	320	9,000	187.13 (167.19-208.80)
T2V	565	16,000	185.86 (170.84-201.83)
B0E	525	15,000	184.21 (168.79-200.66)
V1T	525	15,000	184.21 (168.79-200.66)
L2G	420	12,000	184.21 (167.01-202.70)
N4G	315	9,000	184.21 (164.43-205.72)
T4V	280	8,000	184.21 (163.26-207.10)
B0T	280	8,000	184.21 (163.26-207.10)
L2J	245	7,000	184.21 (161.86-208.78)
P0R	210	6,000	184.21 (160.14-210.88)
E1X	210	6,000	184.21 (160.14-210.88)
T1V	210	6,000	184.21 (160.14-210.88)
E1E	175	5,000	184.21 (157.93-213.62)
B0K	660	19,000	182.83 (169.14-197.32)
N0A	515	15,000	180.70 (165.43-197.00)
N4K	480	14,000	180.45 (164.67-197.34)
E1V	205	6,000	179.82 (156.05-206.20)
N3R	545	16,000	179.28 (164.54-194.98)
V2P	545	16,000	179.28 (164.54-194.98)
V8R	340	10,000	178.95 (160.43-199.02)

T6B	170	5,000	178.95 (153.06-207.96)
S4H	170	5,000	178.95 (153.06-207.96)
C1A	475	14,000	178.57 (162.87-195.38)
L2A	270	8,000	177.63 (157.07-200.13)
V1E	270	8,000	177.63 (157.07-200.13)
N2B	270	8,000	177.63 (157.07-200.13)
C0B	505	15,000	177.19 (162.07-193.34)
L8G	370	11,000	177.03 (159.45-196.02)
S0C	370	11,000	177.03 (159.45-196.02)
E2J	235	7,000	176.69 (154.82-200.79)
T6A	235	7,000	176.69 (154.82-200.79)
V7R	235	7,000	176.69 (154.82-200.79)
R3J	435	13,000	176.11 (159.95-193.47)
L3C	465	14,000	174.81 (159.28-191.45)
V9W	465	14,000	174.81 (159.28-191.45)
C0A	730	22,000	174.64 (162.20-187.78)
V0M	165	5,000	173.68 (148.19-202.30)
L7T	230	7,000	172.93 (151.30-196.79)
V6M	230	7,000	172.93 (151.30-196.79)
L8T	295	9,000	172.51 (153.39-193.37)
V2S	720	22,000	172.25 (159.90-185.30)
L3B	360	11,000	172.25 (154.91-190.99)
V0G	425	13,000	172.06 (156.09-189.23)

V0E	1,045	32,000	171.88 (161.61-182.62)
N0C	260	8,000	171.05 (150.89-193.16)
L2N	485	15,000	170.18 (155.37-186.02)
P7A	420	13,000	170.04 (154.17-187.11)
N8S	355	11,000	169.86 (152.64-188.48)
M9P	290	9,000	169.59 (150.63-190.28)
L0N	450	14,000	169.17 (153.90-185.55)
N6K	450	14,000	169.17 (153.90-185.55)
B0J	770	24,000	168.86 (157.14-181.22)
V1W	385	12,000	168.86 (152.41-186.60)
R0M	320	10,000	168.42 (150.47-187.92)
R7N	160	5,000	168.42 (143.34-196.63)
L7S	160	5,000	168.42 (143.34-196.63)
V8X	350	11,000	167.46 (150.38-185.96)
B0M	285	9,000	166.67 (147.88-187.18)
M4A	190	6,000	166.67 (143.81-192.12)
B3N	190	6,000	166.67 (143.81-192.12)
N0N	630	20,000	165.79 (153.09-179.26)
N0M	1,005	32,000	165.30 (155.23-175.84)
P6B	345	11,000	165.07 (148.11-183.44)
P7E	345	11,000	165.07 (148.11-183.44)
L9H	470	15,000	164.91 (150.34-180.52)
V3A	595	19,000	164.82 (151.84-178.61)

M9B	405	13,000	163.97 (148.39-180.74)
V0K	555	18,000	162.28 (149.06-176.36)
V1N	185	6,000	162.28 (139.74-187.43)
N1A	185	6,000	162.28 (139.74-187.43)
S4P	185	6,000	162.28 (139.74-187.43)
L0P	185	6,000	162.28 (139.74-187.43)
V6N	215	7,000	161.65 (140.77-184.77)
T2S	215	7,000	161.65 (140.77-184.77)
V9L	460	15,000	161.40 (146.99-176.85)
T0B	950	31,000	161.29 (151.20-171.88)
L7R	245	8,000	161.18 (141.63-182.68)
S0E	520	17,000	160.99 (147.45-175.44)
N0P	855	28,000	160.71 (150.12-171.86)
N9E	305	10,000	160.53 (143.01-179.59)
L8V	305	10,000	160.53 (143.01-179.59)
K9V	365	12,000	160.09 (144.08-177.38)
T5R	365	12,000	160.09 (144.08-177.38)
N2L	395	13,000	159.92 (144.54-176.49)
N7T	395	13,000	159.92 (144.54-176.49)
P7B	455	15,000	159.65 (145.31-175.01)
N4S	485	16,000	159.54 (145.65-174.39)
P0H	515	17,000	159.44 (145.97-173.83)
V9T	390	13,000	157.89 (142.61-174.37)

K2H	390	13,000	157.89 (142.61-174.37)
V2C	330	11,000	157.89 (141.32-175.88)
E2K	240	8,000	157.89 (138.55-179.19)
L9R	210	7,000	157.89 (137.26-180.76)
B2G	210	7,000	157.89 (137.26-180.76)
B4A	210	7,000	157.89 (137.26-180.76)
K2A	210	7,000	157.89 (137.26-180.76)
B1H	150	5,000	157.89 (133.64-185.28)
V7P	150	5,000	157.89 (133.64-185.28)
B3M	385	13,000	155.87 (140.69-172.25)
R0K	385	13,000	155.87 (140.69-172.25)
T1A	385	13,000	155.87 (140.69-172.25)
L9A	325	11,000	155.50 (139.05-173.36)
L9G	295	10,000	155.26 (138.05-174.03)
M1M	295	10,000	155.26 (138.05-174.03)
B0W	470	16,000	154.61 (140.94-169.24)
P1B	470	16,000	154.61 (140.94-169.24)
L0K	470	16,000	154.61 (140.94-169.24)
C1N	235	8,000	154.61 (135.47-175.69)
M5P	235	8,000	154.61 (135.47-175.69)
R3P	235	8,000	154.61 (135.47-175.69)
R0L	440	15,000	154.39 (140.30-169.51)
P7C	410	14,000	154.14 (139.57-169.80)

T9A	205	7,000	154.14 (133.76-176.74)
K7H	205	7,000	154.14 (133.76-176.74)
T1J	205	7,000	154.14 (133.76-176.74)
L1G	585	20,000	153.95 (141.72-166.94)
N6J	380	13,000	153.85 (138.76-170.12)
L0L	730	25,000	153.68 (142.74-165.25)
R0C	730	25,000	153.68 (142.74-165.25)
V6G	350	12,000	153.51 (137.85-170.46)
L9L	175	6,000	153.51 (131.61-178.01)
N0E	495	17,000	153.25 (140.05-167.37)
V9A	495	17,000	153.25 (140.05-167.37)
S0L	465	16,000	152.96 (139.37-167.52)
K0C	725	25,000	152.63 (141.72-164.16)
N4X	145	5,000	152.63 (128.80-179.59)
N4W	145	5,000	152.63 (128.80-179.59)
N8M	145	5,000	152.63 (128.80-179.59)
V6L	145	5,000	152.63 (128.80-179.59)
K0L	985	34,000	152.48 (143.10-162.30)
K0E	550	19,000	152.35 (139.89-165.64)
N8Y	260	9,000	152.05 (134.13-171.70)
N7S	375	13,000	151.82 (136.84-167.99)
R2X	230	8,000	151.32 (132.39-172.19)
B4N	230	8,000	151.32 (132.39-172.19)

M2K	230	8,000	151.32 (132.39-172.19)
K9A	315	11,000	150.72 (134.53-168.32)
N5X	315	11,000	150.72 (134.53-168.32)
V9S	200	7,000	150.38 (130.26-172.72)
N9G	200	7,000	150.38 (130.26-172.72)
R3M	285	10,000	150.00 (133.09-168.46)
V0N	1,110	39,000	149.80 (141.11-158.88)
R3R	370	13,000	149.80 (134.92-165.87)
M9A	425	15,000	149.12 (135.28-164.00)
A2H	340	12,000	149.12 (133.69-165.85)
B3L	255	9,000	149.12 (131.38-168.59)
B4V	255	9,000	149.12 (131.38-168.59)
T6C	255	9,000	149.12 (131.38-168.59)
B0H	170	6,000	149.12 (127.55-173.30)
N3L	170	6,000	149.12 (127.55-173.30)
V1Z	170	6,000	149.12 (127.55-173.30)
N0L	650	23,000	148.74 (137.53-160.63)
M9C	480	17,000	148.61 (135.61-162.52)
V4K	310	11,000	148.33 (132.27-165.79)
T3E	535	19,000	148.20 (135.91-161.31)
L8K	450	16,000	148.03 (134.66-162.36)
G6P	450	16,000	148.03 (134.66-162.36)
L0B	225	8,000	148.03 (129.32-168.68)

R2M	505	18,000	147.66 (135.06-161.12)
T6H	420	15,000	147.37 (133.61-162.16)
N5R	335	12,000	146.93 (131.62-163.54)
T0K	725	26,000	146.76 (136.27-157.84)
T5N	195	7,000	146.62 (126.76-168.70)
V1V	195	7,000	146.62 (126.76-168.70)
V7N	195	7,000	146.62 (126.76-168.70)
M8Z	195	7,000	146.62 (126.76-168.70)
P1L	195	7,000	146.62 (126.76-168.70)
L2M	445	16,000	146.38 (133.10-160.64)
G1W	250	9,000	146.20 (128.64-165.49)
R2V	415	15,000	145.61 (131.94-160.32)
R0E	550	20,000	144.74 (132.89-157.35)
R2J	330	12,000	144.74 (129.54-161.23)
R2Y	275	10,000	144.74 (128.13-162.89)
N2H	275	10,000	144.74 (128.13-162.89)
R3N	220	8,000	144.74 (126.24-165.18)
L4R	220	8,000	144.74 (126.24-165.18)
P1A	220	8,000	144.74 (126.24-165.18)
B5A	165	6,000	144.74 (123.49-168.58)
J8H	165	6,000	144.74 (123.49-168.58)
M2L	165	6,000	144.74 (123.49-168.58)
K0G	465	17,000	143.96 (131.17-157.66)

J7Z	355	13,000	143.72 (129.16-159.48)
L6L	355	13,000	143.72 (129.16-159.48)
V9Y	355	13,000	143.72 (129.16-159.48)
V3L	300	11,000	143.54 (127.76-160.74)
G9N	245	9,000	143.27 (125.89-162.38)
V5G	245	9,000	143.27 (125.89-162.38)
T0C	815	30,000	142.98 (133.33-153.15)
E1C	380	14,000	142.86 (128.85-157.97)
K6H	380	14,000	142.86 (128.85-157.97)
R2H	190	7,000	142.86 (123.27-164.68)
E3N	190	7,000	142.86 (123.27-164.68)
N7L	325	12,000	142.54 (127.47-158.91)
B0N	595	22,000	142.34 (131.14-154.26)
E1A	405	15,000	142.11 (128.60-156.64)
K2B	405	15,000	142.11 (128.60-156.64)
V4T	270	10,000	142.11 (125.66-160.11)
E2L	135	5,000	142.11 (119.15-168.20)
N7M	350	13,000	141.70 (127.24-157.35)
T0M	860	32,000	141.45 (132.15-151.23)
V5H	430	16,000	141.45 (128.39-155.47)
J0R	640	24,000	140.35 (129.69-151.66)
R2G	400	15,000	140.35 (126.93-154.80)
R7B	240	9,000	140.35 (123.15-159.28)

K2E	240	9,000	140.35 (123.15-159.28)
J7Y	240	9,000	140.35 (123.15-159.28)
V6R	240	9,000	140.35 (123.15-159.28)
E4P	160	6,000	140.35 (119.45-163.86)
N0G	1,090	41,000	139.92 (131.74-148.48)
L9C	505	19,000	139.89 (127.95-152.64)
A0A	715	27,000	139.38 (129.35-149.98)
S4R	450	17,000	139.32 (126.74-152.81)
B2W	370	14,000	139.10 (125.28-154.02)
G6L	185	7,000	139.10 (119.78-160.65)
A0N	185	7,000	139.10 (119.78-160.65)
B4H	185	7,000	139.10 (119.78-160.65)
T1K	475	18,000	138.89 (126.68-151.96)
N6C	395	15,000	138.60 (125.26-152.96)
K1J	315	12,000	138.16 (123.32-154.29)
L6J	315	12,000	138.16 (123.32-154.29)
A0C	210	8,000	138.16 (120.10-158.16)
E1B	210	8,000	138.16 (120.10-158.16)
K6J	210	8,000	138.16 (120.10-158.16)
G9T	210	8,000	138.16 (120.10-158.16)
R0G	550	21,000	137.84 (126.56-149.86)
V2B	445	17,000	137.77 (125.27-151.19)
B0P	575	22,000	137.56 (126.54-149.28)

V2R	470	18,000	137.43 (125.28-150.43)
B3H	235	9,000	137.43 (120.42-156.17)
V6J	235	9,000	137.43 (120.42-156.17)
M1R	365	14,000	137.22 (123.50-152.04)
V9G	130	5,000	136.84 (114.33-162.49)
T4H	130	5,000	136.84 (114.33-162.49)
T0E	545	21,000	136.59 (125.36-148.56)
M9R	415	16,000	136.51 (123.69-150.30)
L3M	285	11,000	136.36 (120.99-153.15)
M1N	285	11,000	136.36 (120.99-153.15)
T3B	440	17,000	136.22 (123.79-149.57)
K0J	440	17,000	136.22 (123.79-149.57)
M2R	465	18,000	135.96 (123.89-148.90)
K9H	310	12,000	135.96 (121.25-151.97)
L5E	155	6,000	135.96 (115.40-159.13)
K6A	155	6,000	135.96 (115.40-159.13)
L6K	155	6,000	135.96 (115.40-159.13)
J3R	155	6,000	135.96 (115.40-159.13)
L8S	180	7,000	135.34 (116.29-156.62)
V2Z	180	7,000	135.34 (116.29-156.62)
M5N	180	7,000	135.34 (116.29-156.62)
L1H	410	16,000	134.87 (122.13-148.58)
J2X	205	8,000	134.87 (117.04-154.65)

V1L	205	8,000	134.87 (117.04-154.65)
B1A	205	8,000	134.87 (117.04-154.65)
V4W	230	9,000	134.50 (117.68-153.06)
V1B	230	9,000	134.50 (117.68-153.06)
L6W	280	11,000	133.97 (118.74-150.62)
M5M	280	11,000	133.97 (118.74-150.62)
V7L	280	11,000	133.97 (118.74-150.62)
N5W	305	12,000	133.77 (119.18-149.66)
R7A	305	12,000	133.77 (119.18-149.66)
T2J	635	25,000	133.68 (123.49-144.50)
E3A	330	13,000	133.60 (119.58-148.82)
J3P	330	13,000	133.60 (119.58-148.82)
K1K	355	14,000	133.46 (119.94-148.09)
N5A	380	15,000	133.33 (120.26-147.44)
T5E	380	15,000	133.33 (120.26-147.44)
K1G	405	16,000	133.22 (120.56-146.85)
P6A	430	17,000	133.13 (120.84-146.32)
A0G	505	20,000	132.89 (121.56-145.01)
R3T	525	21,000	131.58 (120.56-143.33)
P0T	475	19,000	131.58 (120.01-143.96)
J3B	350	14,000	131.58 (118.15-146.11)
G1H	325	13,000	131.58 (117.66-146.69)
V5C	300	12,000	131.58 (117.11-147.34)

A0B	300	12,000	131.58 (117.11-147.34)
B3A	250	10,000	131.58 (115.77-148.94)
V5J	225	9,000	131.58 (114.95-149.94)
M3L	200	8,000	131.58 (113.97-151.13)
E2E	200	8,000	131.58 (113.97-151.13)
K1L	200	8,000	131.58 (113.97-151.13)
M4G	200	8,000	131.58 (113.97-151.13)
T5L	200	8,000	131.58 (113.97-151.13)
N9Y	175	7,000	131.58 (112.81-152.58)
V6H	175	7,000	131.58 (112.81-152.58)
V2T	595	24,000	130.48 (120.21-141.40)
G8T	370	15,000	129.82 (116.93-143.75)
V1M	320	13,000	129.55 (115.75-144.56)
P3A	295	12,000	129.39 (115.04-145.03)
M5R	270	11,000	129.19 (114.24-145.55)
L4A	270	11,000	129.19 (114.24-145.55)
T1H	270	11,000	129.19 (114.24-145.55)
L0E	245	10,000	128.95 (113.31-146.15)
M1H	245	10,000	128.95 (113.31-146.15)
A0H	245	10,000	128.95 (113.31-146.15)
P6C	245	10,000	128.95 (113.31-146.15)
V3J	465	19,000	128.81 (117.37-141.07)
L7L	440	18,000	128.65 (116.91-141.26)

M1T	365	15,000	128.07 (115.27-141.91)
B2N	340	14,000	127.82 (114.59-142.15)
L0G	485	20,000	127.63 (116.52-139.51)
N8H	315	13,000	127.53 (113.83-142.42)
K2C	315	13,000	127.53 (113.83-142.42)
J2G	435	18,000	127.19 (115.52-139.73)
N1G	290	12,000	127.19 (112.97-142.71)
V9R	290	12,000	127.19 (112.97-142.71)
N0R	505	21,000	126.57 (115.77-138.10)
K9J	505	21,000	126.57 (115.77-138.10)
K7M	505	21,000	126.57 (115.77-138.10)
V1X	360	15,000	126.32 (113.60-140.06)
A0E	335	14,000	125.94 (112.81-140.17)
M2M	335	14,000	125.94 (112.81-140.17)
L3V	475	20,000	125.00 (114.01-136.76)
R2K	380	16,000	125.00 (112.75-138.22)
L0R	900	38,000	124.65 (116.64-133.07)
N0B	875	37,000	124.47 (116.36-132.99)
S0K	875	37,000	124.47 (116.36-132.99)
T6J	520	22,000	124.40 (113.94-135.57)
T0L	775	33,000	123.60 (115.05-132.62)
S0J	560	24,000	122.81 (112.84-133.41)

† Number of cases was rounded to a multiple of 5 as per SSHRC/Statistics Canada regulations.

Supplementary Table 5. Overall prostate cancer incidence rates by Forward Sortation Area (FSA) between 1992 and 2010. Incidence rates are expressed per 100,000 men per year. Only FSAs with statistically significant lower incidence rates compared to the national average are included in this table.

**FSA**	**Cases †**	**Male Population (rounded 1,000)**	**Incidence Rate (95% CI)**
T0H	790	40,000	103.95 (96.82-111.46)
J0K	1,285	67,000	100.94 (95.50-106.62)
V0J	555	29,000	100.73 (92.52-109.47)
M1K	440	23,000	100.69 (91.50-110.55)
G0L	610	32,000	100.33 (92.52-108.62)
L4L	495	26,000	100.20 (91.57-109.43)
A0K	340	18,000	99.42 (89.13-110.56)
V7E	320	17,000	99.07 (88.51-110.54)
M3C	320	17,000	99.07 (88.51-110.54)
G6V	320	17,000	99.07 (88.51-110.54)
L4G	395	21,000	99.00 (89.47-109.26)
L1C	300	16,000	98.68 (87.83-110.51)
M8V	280	15,000	98.25 (87.07-110.45)
N5V	280	15,000	98.25 (87.07-110.45)
V7C	335	18,000	97.95 (87.74-109.02)
J6E	335	18,000	97.95 (87.74-109.02)
J4B	335	18,000	97.95 (87.74-109.02)

G0M	335	18,000	97.95 (87.74-109.02)
M1J	315	17,000	97.52 (87.05-108.91)
L8E	315	17,000	97.52 (87.05-108.91)
R0A	370	20,000	97.37 (87.70-107.81)
V2X	425	23,000	97.25 (88.23-106.95)
G0A	885	48,000	97.04 (90.75-103.65)
L7G	350	19,000	96.95 (87.06-107.66)
T0G	405	22,000	96.89 (87.68-106.80)
J0B	605	33,000	96.49 (88.95-104.50)
K2G	385	21,000	96.49 (87.09-106.63)
H4L	275	15,000	96.49 (85.42-108.60)
K1N	220	12,000	96.49 (84.16-110.12)
V6E	220	12,000	96.49 (84.16-110.12)
J0L	710	39,000	95.82 (88.90-103.13)
V6K	200	11,000	95.69 (82.89-109.91)
M6E	345	19,000	95.57 (85.75-106.20)
H1H	290	16,000	95.39 (84.73-107.03)
L6S	470	26,000	95.14 (86.73-104.14)
P0L	235	13,000	95.14 (83.37-108.12)
H7N	325	18,000	95.03 (84.98-105.94)
L5A	415	23,000	94.97 (86.05-104.56)
L5L	415	23,000	94.97 (86.05-104.56)
J6A	305	17,000	94.43 (84.13-105.64)

J0E	430	24,000	94.30 (85.59-103.65)
R3G	215	12,000	94.30 (82.11-107.78)
V7A	215	12,000	94.30 (82.11-107.78)
M6M	340	19,000	94.18 (84.44-104.75)
K1C	340	19,000	94.18 (84.44-104.75)
G1V	160	9,000	93.57 (79.63-109.24)
H4V	160	9,000	93.57 (79.63-109.24)
T8L	160	9,000	93.57 (79.63-109.24)
R3E	160	9,000	93.57 (79.63-109.24)
M4C	390	22,000	93.30 (84.27-103.04)
H8N	230	13,000	93.12 (81.47-105.96)
M6B	230	13,000	93.12 (81.47-105.96)
J4L	265	15,000	92.98 (82.12-104.88)
G8B	210	12,000	92.11 (80.07-105.44)
H3X	175	10,000	92.11 (78.96-106.81)
M6R	175	10,000	92.11 (78.96-106.81)
V3Y	140	8,000	92.11 (77.48-108.69)
L9Z	140	8,000	92.11 (77.48-108.69)
V2E	105	6,000	92.11 (75.33-111.50)
E5N	105	6,000	92.11 (75.33-111.50)
G0S	610	35,000	91.73 (84.59-99.31)
H1G	400	23,000	91.53 (82.78-100.96)
G2E	190	11,000	90.91 (78.44-104.80)

M6N	345	20,000	90.79 (81.46-100.89)
H3L	155	9,000	90.64 (76.94-106.09)
G5R	155	9,000	90.64 (76.94-106.09)
K2K	155	9,000	90.64 (76.94-106.09)
V5N	275	16,000	90.46 (80.08-101.81)
H4E	240	14,000	90.23 (79.17-102.39)
B3T	120	7,000	90.23 (74.81-107.89)
L8M	120	7,000	90.23 (74.81-107.89)
L1V	410	24,000	89.91 (81.42-99.05)
V2M	205	12,000	89.91 (78.02-103.10)
T8V	290	17,000	89.78 (79.75-100.73)
N1R	340	20,000	89.47 (80.21-99.51)
L5C	255	15,000	89.47 (78.83-101.16)
J4J	255	15,000	89.47 (78.83-101.16)
T2C	170	10,000	89.47 (76.53-103.98)
H3Y	85	5,000	89.47 (71.47-110.64)
J1A	85	5,000	89.47 (71.47-110.64)
K9K	85	5,000	89.47 (71.47-110.64)
L9N	85	5,000	89.47 (71.47-110.64)
T1G	85	5,000	89.47 (71.47-110.64)
V4X	85	5,000	89.47 (71.47-110.64)
G6X	85	5,000	89.47 (71.47-110.64)
V6T	85	5,000	89.47 (71.47-110.64)

G0J	305	18,000	89.18 (79.45-99.77)
T2T	220	13,000	89.07 (77.69-101.65)
M4P	135	8,000	88.82 (74.47-105.12)
M6A	135	8,000	88.82 (74.47-105.12)
H1P	135	8,000	88.82 (74.47-105.12)
V5M	185	11,000	88.52 (76.22-102.23)
J6J	185	11,000	88.52 (76.22-102.23)
T6E	185	11,000	88.52 (76.22-102.23)
L3R	470	28,000	88.35 (80.54-96.71)
H7W	300	18,000	87.72 (78.07-98.23)
V3C	300	18,000	87.72 (78.07-98.23)
J4K	200	12,000	87.72 (75.98-100.76)
A1N	200	12,000	87.72 (75.98-100.76)
N2N	200	12,000	87.72 (75.98-100.76)
J2C	200	12,000	87.72 (75.98-100.76)
L4P	200	12,000	87.72 (75.98-100.76)
M3M	200	12,000	87.72 (75.98-100.76)
G7B	150	9,000	87.72 (74.24-102.93)
T7E	100	6,000	87.72 (71.37-106.69)
G4W	100	6,000	87.72 (71.37-106.69)
V3N	265	16,000	87.17 (76.99-98.32)
H1X	215	13,000	87.04 (75.80-99.49)
L4C	545	33,000	86.92 (79.78-94.54)

J6K	165	10,000	86.84 (74.10-101.15)
H1R	230	14,000	86.47 (75.65-98.39)
T7X	230	14,000	86.47 (75.65-98.39)
G2A	115	7,000	86.47 (71.39-103.79)
K7R	115	7,000	86.47 (71.39-103.79)
L9M	115	7,000	86.47 (71.39-103.79)
V8J	115	7,000	86.47 (71.39-103.79)
G2B	180	11,000	86.12 (74.00-99.67)
H1N	180	11,000	86.12 (74.00-99.67)
M6J	245	15,000	85.96 (75.54-97.43)
M4K	245	15,000	85.96 (75.54-97.43)
R2C	260	16,000	85.53 (75.45-96.58)
V2N	260	16,000	85.53 (75.45-96.58)
H2C	130	8,000	85.53 (71.46-101.56)
J6T	130	8,000	85.53 (71.46-101.56)
V3B	355	22,000	84.93 (76.32-94.24)
M6P	290	18,000	84.80 (75.32-95.14)
V5W	145	9,000	84.80 (71.56-99.77)
V8T	145	9,000	84.80 (71.56-99.77)
S0M	370	23,000	84.67 (76.26-93.75)
R2W	225	14,000	84.59 (73.89-96.39)
G5L	225	14,000	84.59 (73.89-96.39)
V0C	225	14,000	84.59 (73.89-96.39)

J3L	305	19,000	84.49 (75.27-94.52)
H1A	240	15,000	84.21 (73.89-95.57)
M4S	160	10,000	84.21 (71.67-98.32)
G7S	160	10,000	84.21 (71.67-98.32)
V2K	160	10,000	84.21 (71.67-98.32)
V6A	160	10,000	84.21 (71.67-98.32)
J8B	80	5,000	84.21 (66.77-104.81)
T7V	80	5,000	84.21 (66.77-104.81)
L8R	80	5,000	84.21 (66.77-104.81)
M5B	80	5,000	84.21 (66.77-104.81)
T8R	80	5,000	84.21 (66.77-104.81)
T3G	335	21,000	83.96 (75.21-93.45)
T5A	255	16,000	83.88 (73.90-94.83)
L3Z	175	11,000	83.73 (71.79-97.10)
H9R	175	11,000	83.73 (71.79-97.10)
J0H	445	28,000	83.65 (76.05-91.79)
L7M	270	17,000	83.59 (73.92-94.18)
R2P	190	12,000	83.33 (71.90-96.06)
J6N	95	6,000	83.33 (67.42-101.87)
T1W	95	6,000	83.33 (67.42-101.87)
G0C	410	26,000	83.00 (75.16-91.43)
L6V	315	20,000	82.89 (73.99-92.57)
V5R	315	20,000	82.89 (73.99-92.57)

V4N	330	21,000	82.71 (74.02-92.13)
J6W	220	14,000	82.71 (72.14-94.39)
M1L	220	14,000	82.71 (72.14-94.39)
N6E	220	14,000	82.71 (72.14-94.39)
K7P	110	7,000	82.71 (67.97-99.68)
P3B	110	7,000	82.71 (67.97-99.68)
L8N	110	7,000	82.71 (67.97-99.68)
H1K	235	15,000	82.46 (72.25-93.70)
N2T	125	8,000	82.24 (68.45-97.98)
B4E	125	8,000	82.24 (68.45-97.98)
T9V	125	8,000	82.24 (68.45-97.98)
T5T	390	25,000	82.11 (74.16-90.67)
L6H	390	25,000	82.11 (74.16-90.67)
J0P	265	17,000	82.04 (72.46-92.54)
J1G	140	9,000	81.87 (68.87-96.61)
M9V	435	28,000	81.77 (74.26-89.82)
K1Z	155	10,000	81.58 (69.24-95.48)
J3H	170	11,000	81.34 (69.57-94.53)
V5V	170	11,000	81.34 (69.57-94.53)
L1E	170	11,000	81.34 (69.57-94.53)
L9T	355	23,000	81.24 (73.00-90.14)
T6R	185	12,000	81.14 (69.87-93.71)
T2B	185	12,000	81.14 (69.87-93.71)

L4N	585	38,000	81.02 (74.59-87.87)
V5X	200	13,000	80.97 (70.14-93.00)
H8R	215	14,000	80.83 (70.38-92.38)
M1B	460	30,000	80.70 (73.49-88.42)
H1L	245	16,000	80.59 (70.82-91.34)
M4J	260	17,000	80.50 (71.01-90.90)
V3W	580	38,000	80.33 (73.93-87.14)
M6H	345	23,000	78.95 (70.84-87.73)
M3N	315	21,000	78.95 (70.47-88.17)
T6K	225	15,000	78.95 (68.97-89.96)
M4L	225	15,000	78.95 (68.97-89.96)
L7E	210	14,000	78.95 (68.63-90.38)
J9X	210	14,000	78.95 (68.63-90.38)
J8Y	180	12,000	78.95 (67.84-91.36)
S7N	180	12,000	78.95 (67.84-91.36)
J6S	165	11,000	78.95 (67.36-91.95)
H9H	165	11,000	78.95 (67.36-91.95)
T4B	150	10,000	78.95 (66.82-92.64)
J1E	135	9,000	78.95 (66.19-93.44)
G0V	135	9,000	78.95 (66.19-93.44)
H4R	120	8,000	78.95 (65.46-94.40)
M4H	120	8,000	78.95 (65.46-94.40)
H4J	120	8,000	78.95 (65.46-94.40)

B2T	105	7,000	78.95 (64.57-95.57)
V0A	105	7,000	78.95 (64.57-95.57)
R3B	90	6,000	78.95 (63.48-97.04)
T2R	90	6,000	78.95 (63.48-97.04)
G5C	90	6,000	78.95 (63.48-97.04)
V6Z	90	6,000	78.95 (63.48-97.04)
K4K	90	6,000	78.95 (63.48-97.04)
V5Y	90	6,000	78.95 (63.48-97.04)
A1W	75	5,000	78.95 (62.10-98.96)
B0A	75	5,000	78.95 (62.10-98.96)
E7M	75	5,000	78.95 (62.10-98.96)
P9A	75	5,000	78.95 (62.10-98.96)
V0T	75	5,000	78.95 (62.10-98.96)
V4R	75	5,000	78.95 (62.10-98.96)
G8H	75	5,000	78.95 (62.10-98.96)
J2H	75	5,000	78.95 (62.10-98.96)
T5X	250	17,000	77.40 (68.10-87.61)
L4J	455	31,000	77.25 (70.31-84.68)
J2B	205	14,000	77.07 (66.88-88.37)
N3C	160	11,000	76.56 (65.15-89.38)
V6X	160	11,000	76.56 (65.15-89.38)
H2G	160	11,000	76.56 (65.15-89.38)
L4T	290	20,000	76.32 (67.78-85.62)

H2R	145	10,000	76.32 (64.40-89.80)
V8G	145	10,000	76.32 (64.40-89.80)
H4B	130	9,000	76.02 (63.52-90.27)
J4W	130	9,000	76.02 (63.52-90.27)
L8L	245	17,000	75.85 (66.65-85.97)
J4X	115	8,000	75.66 (62.46-90.82)
L6Y	430	30,000	75.44 (68.48-82.92)
V3V	300	21,000	75.19 (66.92-84.20)
J1H	200	14,000	75.19 (65.13-86.36)
J9L	100	7,000	75.19 (61.18-91.45)
N2P	100	7,000	75.19 (61.18-91.45)
T8H	100	7,000	75.19 (61.18-91.45)
E2V	100	7,000	75.19 (61.18-91.45)
L4K	100	7,000	75.19 (61.18-91.45)
J0Z	285	20,000	75.00 (66.54-84.23)
T2X	185	13,000	74.90 (64.49-86.50)
H2L	185	13,000	74.90 (64.49-86.50)
H2E	170	12,000	74.56 (63.77-86.65)
L8W	170	12,000	74.56 (63.77-86.65)
G5Y	170	12,000	74.56 (63.77-86.65)
G6E	85	6,000	74.56 (59.56-92.20)
G4T	85	6,000	74.56 (59.56-92.20)
T2A	410	29,000	74.41 (67.38-81.97)

J0X	310	22,000	74.16 (66.14-82.90)
J7P	155	11,000	74.16 (62.95-86.80)
G1C	225	16,000	74.01 (64.66-84.34)
V2W	70	5,000	73.68 (57.44-93.10)
G3L	70	5,000	73.68 (57.44-93.10)
J3Y	390	28,000	73.31 (66.21-80.96)
V5P	195	14,000	73.31 (63.38-84.35)
J0N	320	23,000	73.23 (65.42-81.71)
T2Y	250	18,000	73.10 (64.32-82.74)
T4C	125	9,000	73.10 (60.85-87.09)
G0G	125	9,000	73.10 (60.85-87.09)
M4Y	180	13,000	72.87 (62.62-84.33)
J5R	235	17,000	72.76 (63.75-82.68)
G0W	290	21,000	72.68 (64.56-81.55)
H1E	275	20,000	72.37 (64.07-81.45)
H1Y	165	12,000	72.37 (61.75-84.29)
L9S	165	12,000	72.37 (61.75-84.29)
H4N	165	12,000	72.37 (61.75-84.29)
E0E	165	12,000	72.37 (61.75-84.29)
H9G	110	8,000	72.37 (59.48-87.22)
L1X	110	8,000	72.37 (59.48-87.22)
G7A	110	8,000	72.37 (59.48-87.22)
H7P	205	15,000	71.93 (62.42-82.48)

J0C	150	11,000	71.77 (60.74-84.22)
J2W	150	11,000	71.77 (60.74-84.22)
T4R	150	11,000	71.77 (60.74-84.22)
V1J	150	11,000	71.77 (60.74-84.22)
G0K	95	7,000	71.43 (57.79-87.32)
J7A	95	7,000	71.43 (57.79-87.32)
G7J	95	7,000	71.43 (57.79-87.32)
H3K	95	7,000	71.43 (57.79-87.32)
J7R	230	17,000	71.21 (62.30-81.03)
M1V	365	27,000	71.15 (64.04-78.84)
H9B	135	10,000	71.05 (59.57-84.10)
J4Z	135	10,000	71.05 (59.57-84.10)
V5L	135	10,000	71.05 (59.57-84.10)
K2L	135	10,000	71.05 (59.57-84.10)
L6Z	215	16,000	70.72 (61.59-80.84)
S7M	200	15,000	70.18 (60.79-80.60)
H4A	160	12,000	70.18 (59.72-81.93)
H7R	160	12,000	70.18 (59.72-81.93)
J4T	120	9,000	70.18 (58.18-83.91)
H8Y	120	9,000	70.18 (58.18-83.91)
A1L	80	6,000	70.18 (55.64-87.34)
J4M	80	6,000	70.18 (55.64-87.34)
T7A	80	6,000	70.18 (55.64-87.34)

G0H	80	6,000	70.18 (55.64-87.34)
L5B	305	23,000	69.79 (62.18-78.08)
M5A	225	17,000	69.66 (60.85-79.38)
M6K	225	17,000	69.66 (60.85-79.38)
L6X	250	19,000	69.25 (60.93-78.39)
J4Y	105	8,000	69.08 (56.50-83.62)
T1R	105	8,000	69.08 (56.50-83.62)
T3L	130	10,000	68.42 (57.17-81.24)
B3G	65	5,000	68.42 (52.81-87.21)
J6R	65	5,000	68.42 (52.81-87.21)
N2V	65	5,000	68.42 (52.81-87.21)
G2L	65	5,000	68.42 (52.81-87.21)
G4Z	65	5,000	68.42 (52.81-87.21)
J8X	65	5,000	68.42 (52.81-87.21)
V6V	65	5,000	68.42 (52.81-87.21)
S7L	245	19,000	67.87 (59.63-76.92)
J7J	90	7,000	67.67 (54.41-83.18)
H2P	90	7,000	67.67 (54.41-83.18)
L8J	115	9,000	67.25 (55.52-80.73)
R2R	115	9,000	67.25 (55.52-80.73)
H2A	115	9,000	67.25 (55.52-80.73)
J6Z	115	9,000	67.25 (55.52-80.73)
H3H	115	9,000	67.25 (55.52-80.73)

T5H	165	13,000	66.80 (57.00-77.81)
K4A	190	15,000	66.67 (57.52-76.85)
J0S	215	17,000	66.56 (57.96-76.08)
H4G	175	14,000	65.79 (56.40-76.29)
H1W	175	14,000	65.79 (56.40-76.29)
K1T	150	12,000	65.79 (55.68-77.20)
K1R	125	10,000	65.79 (54.76-78.39)
H7X	125	10,000	65.79 (54.76-78.39)
V6B	100	8,000	65.79 (53.53-80.02)
H7A	100	8,000	65.79 (53.53-80.02)
L1P	75	6,000	65.79 (51.75-82.47)
K7K	185	15,000	64.91 (55.90-74.97)
T6L	320	26,000	64.78 (57.87-72.28)
J7K	135	11,000	64.59 (54.16-76.45)
V5T	135	11,000	64.59 (54.16-76.45)
T1Y	330	27,000	64.33 (57.57-71.66)
H7K	110	9,000	64.33 (52.87-77.53)
H8Z	85	7,000	63.91 (51.05-79.03)
J9T	85	7,000	63.91 (51.05-79.03)
G1B	85	7,000	63.91 (51.05-79.03)
R6W	85	7,000	63.91 (51.05-79.03)
E0G	315	26,000	63.77 (56.92-71.21)
M3J	145	12,000	63.60 (53.67-74.83)

L1T	205	17,000	63.47 (55.08-72.78)
E0J	240	20,000	63.16 (55.42-71.67)
H7L	180	15,000	63.16 (54.27-73.09)
L4Z	180	15,000	63.16 (54.27-73.09)
H1V	120	10,000	63.16 (52.36-75.52)
H3J	60	5,000	63.16 (48.20-81.30)
T2Z	215	18,000	62.87 (54.74-71.85)
H7T	95	8,000	62.50 (50.57-76.40)
L5N	450	38,000	62.33 (56.70-68.36)
J6X	130	11,000	62.20 (51.97-73.86)
Y1A	130	11,000	62.20 (51.97-73.86)
E0H	130	11,000	62.20 (51.97-73.86)
E0A	495	42,000	62.03 (56.69-67.74)
J9P	165	14,000	62.03 (52.93-72.25)
H2V	165	14,000	62.03 (52.93-72.25)
L5M	400	34,000	61.92 (56.00-68.30)
J1N	140	12,000	61.40 (51.65-72.46)
L6C	140	12,000	61.40 (51.65-72.46)
M5T	105	9,000	61.40 (50.22-74.33)
H4M	70	6,000	61.40 (47.87-77.58)
J2J	70	6,000	61.40 (47.87-77.58)
G9H	70	6,000	61.40 (47.87-77.58)
H3T	70	6,000	61.40 (47.87-77.58)

K2J	220	19,000	60.94 (53.15-69.55)
H2X	115	10,000	60.53 (49.97-72.65)
T4S	80	7,000	60.15 (47.70-74.86)
G8L	80	7,000	60.15 (47.70-74.86)
G0B	80	7,000	60.15 (47.70-74.86)
H9J	125	11,000	59.81 (49.78-71.26)
N2E	170	15,000	59.65 (51.02-69.32)
J5Z	135	12,000	59.21 (49.64-70.08)
H9A	90	8,000	59.21 (47.61-72.78)
L3X	145	13,000	58.70 (49.54-69.07)
J7G	100	9,000	58.48 (47.58-71.13)
H2K	155	14,000	58.27 (49.46-68.20)
V3E	155	14,000	58.27 (49.46-68.20)
H3W	155	14,000	58.27 (49.46-68.20)
L6M	210	19,000	58.17 (50.57-66.59)
J5W	110	10,000	57.89 (47.58-69.78)
S4X	110	10,000	57.89 (47.58-69.78)
J7L	55	5,000	57.89 (43.61-75.36)
H1J	55	5,000	57.89 (43.61-75.36)
H7H	55	5,000	57.89 (43.61-75.36)
J8E	55	5,000	57.89 (43.61-75.36)
G8K	55	5,000	57.89 (43.61-75.36)
L1K	120	11,000	57.42 (47.60-68.66)

J0Y	195	18,000	57.02 (49.30-65.61)
P8N	65	6,000	57.02 (44.00-72.67)
N1T	65	6,000	57.02 (44.00-72.67)
L1R	150	14,000	56.39 (47.73-66.17)
J2K	75	7,000	56.39 (44.36-70.69)
L4B	160	15,000	56.14 (47.78-65.54)
G3A	85	8,000	55.92 (44.67-69.15)
J7N	85	8,000	55.92 (44.67-69.15)
H1Z	180	17,000	55.73 (47.88-64.49)
K2M	95	9,000	55.56 (44.95-67.91)
T5Z	95	9,000	55.56 (44.95-67.91)
J7V	295	28,000	55.45 (49.30-62.15)
M4X	105	10,000	55.26 (45.20-66.90)
J5A	125	12,000	54.82 (45.64-65.32)
J3E	125	12,000	54.82 (45.64-65.32)
J7C	165	16,000	54.28 (46.31-63.22)
L5R	165	16,000	54.28 (46.31-63.22)
H3S	165	16,000	54.28 (46.31-63.22)
T3K	260	26,000	52.63 (46.43-59.43)
L7A	150	15,000	52.63 (44.55-61.76)
L4E	120	12,000	52.63 (43.64-62.93)
M4M	120	12,000	52.63 (43.64-62.93)
T4P	110	11,000	52.63 (43.26-63.44)

J3X	100	10,000	52.63 (42.82-64.01)
J8L	80	8,000	52.63 (41.73-65.50)
P3P	70	7,000	52.63 (41.03-66.50)
J3N	70	7,000	52.63 (41.03-66.50)
T4T	70	7,000	52.63 (41.03-66.50)
G3E	60	6,000	52.63 (40.16-67.75)
R5G	50	5,000	52.63 (39.06-69.39)
L3S	225	23,000	51.49 (44.98-58.67)
H2S	125	13,000	50.61 (42.13-60.30)
T1S	115	12,000	50.44 (41.64-60.54)
J8T	180	19,000	49.86 (42.84-57.70)
J8P	150	16,000	49.34 (41.76-57.90)
T9M	75	8,000	49.34 (38.81-61.85)
N3A	65	7,000	48.87 (37.72-62.29)
H4C	65	7,000	48.87 (37.72-62.29)
K8H	65	7,000	48.87 (37.72-62.29)
H2J	120	13,000	48.58 (40.28-58.09)
H2W	55	6,000	48.25 (36.35-62.80)
G3G	45	5,000	47.37 (34.55-63.38)
J2R	45	5,000	47.37 (34.55-63.38)
E0B	260	29,000	47.19 (41.63-53.29)
G6Z	80	9,000	46.78 (37.10-58.23)
J9H	115	13,000	46.56 (38.44-55.89)

L5V	165	19,000	45.71 (39.00-53.24)
J5Y	95	11,000	45.45 (36.78-55.57)
E0C	155	18,000	45.32 (38.47-53.04)
G3K	60	7,000	45.11 (34.43-58.07)
G4X	60	7,000	45.11 (34.43-58.07)
H2H	60	7,000	45.11 (34.43-58.07)
T5Y	110	13,000	44.53 (36.60-53.68)
L4S	100	12,000	43.86 (35.69-53.35)
H1C	50	6,000	43.86 (32.55-57.82)
J6Y	50	6,000	43.86 (32.55-57.82)
T7S	50	6,000	43.86 (32.55-57.82)
H9C	50	6,000	43.86 (32.55-57.82)
J5K	50	6,000	43.86 (32.55-57.82)
S9V	50	6,000	43.86 (32.55-57.82)
L5W	50	6,000	43.86 (32.55-57.82)
L6A	190	23,000	43.48 (37.52-50.12)
J5L	65	8,000	42.76 (33.00-54.51)
G6J	40	5,000	42.11 (30.08-57.34)
L1Z	40	5,000	42.11 (30.08-57.34)
J8Z	55	7,000	41.35 (31.15-53.83)
L6R	180	23,000	41.19 (35.39-47.67)
H3N	125	16,000	41.12 (34.23-48.99)
L4H	115	15,000	40.35 (33.31-48.44)

T9H	105	14,000	39.47 (32.29-47.79)
P0V	75	10,000	39.47 (31.05-49.48)
H2T	60	8,000	39.47 (30.12-50.81)
T6T	45	6,000	39.47 (28.79-52.82)
X1A	65	9,000	38.01 (29.34-48.45)
R0B	150	21,000	37.59 (31.82-44.11)
P9N	50	7,000	37.59 (27.90-49.56)
L1M	50	7,000	37.59 (27.90-49.56)
R8N	50	7,000	37.59 (27.90-49.56)
X0E	85	12,000	37.28 (29.78-46.10)
T8W	35	5,000	36.84 (25.66-51.24)
E0K	55	8,000	36.18 (27.26-47.10)
M5V	55	8,000	36.18 (27.26-47.10)
L6E	55	8,000	36.18 (27.26-47.10)
J5J	40	6,000	35.09 (25.07-47.78)
J7T	65	10,000	34.21 (26.40-43.60)
J9A	45	7,000	33.83 (24.68-45.27)
T3J	145	23,000	33.18 (28.00-39.04)
J7M	60	10,000	31.58 (24.10-40.65)
A0P	35	6,000	30.70 (21.38-42.70)
J9J	40	7,000	30.08 (21.49-40.95)
L6P	85	16,000	27.96 (22.33-34.57)
J5M	40	8,000	26.32 (18.80-35.83)

J8N	25	5,000	26.32 (17.03-38.85)
T9K	40	9,000	23.39 (16.71-31.85)
V9Z	35	8,000	23.03 (16.04-32.02)
E0L	35	8,000	23.03 (16.04-32.02)
J5T	25	6,000	21.93 (14.19-32.37)
J8V	40	10,000	21.05 (15.04-28.67)
J8M	20	5,000	21.05 (12.86-32.51)
J5C	30	8,000	19.74 (13.32-28.18)
B6L	20	6,000	17.54 (10.72-27.10)
J8R	35	11,000	16.75 (11.66-23.29)
J0M	20	9,000	11.70 (7.14-18.06)
X0A	15	8,000	9.87 (5.52-16.28)

† Number of cases was rounded to a multiple of 5 as per SSHRC/Statistics Canada regulations.

Supplementary Table 6. Overall prostate cancer mortality rates by Forward Sortation Area (FSA) between 1992 and 2010. Mortality rates are expressed per 100,000 men per year. Only FSAs with statistically significant higher mortality rates compared to the national average are included in this table.

FSA	Deaths †	Male Population (rounded to 1,000)	Incidence Rate (95% CI)
S3N	70	8,000	79.55 (62.01-100.50)
S0A	235	28,000	76.30 (66.85-86.70)
H9X	40	5,000	72.73 (51.96-99.03)
R3J	100	13,000	69.93 (56.90-85.05)
V8L	75	10,000	68.18 (53.63-85.47)
V1Y	105	15,000	63.64 (52.05-77.04)
H3Z	35	5,000	63.64 (44.33-88.50)
R0J	90	13,000	62.94 (50.61-77.36)
R0L	100	15,000	60.61 (49.31-73.71)
P5A	40	6,000	60.61 (43.30-82.53)
S0L	105	16,000	59.66 (48.80-72.22)
R0M	65	10,000	59.09 (45.61-75.32)
V9K	45	7,000	58.44 (42.63-78.20)
T1J	45	7,000	58.44 (42.63-78.20)
S9A	45	7,000	58.44 (42.63-78.20)
V7V	45	7,000	58.44 (42.63-78.20)
M4N	45	7,000	58.44 (42.63-78.20)

V9M	45	7,000	58.44 (42.63-78.20)
S4S	100	16,000	56.82 (46.23-69.11)
K9V	75	12,000	56.82 (44.69-71.22)
V1E	50	8,000	56.82 (42.17-74.91)
S6H	85	14,000	55.19 (44.09-68.25)
G1S	60	10,000	54.55 (41.62-70.21)
V9P	60	10,000	54.55 (41.62-70.21)
P1P	30	5,000	54.55 (36.80-77.87)
R7N	30	5,000	54.55 (36.80-77.87)
E1E	30	5,000	54.55 (36.80-77.87)
S4H	30	5,000	54.55 (36.80-77.87)
V2A	100	17,000	53.48 (43.51-65.04)
S0H	70	12,000	53.03 (41.34-67.00)
T1V	35	6,000	53.03 (36.94-73.75)
E1X	35	6,000	53.03 (36.94-73.75)
V1N	35	6,000	53.03 (36.94-73.75)
V0H	145	25,000	52.73 (44.49-62.04)
P1H	40	7,000	51.95 (37.11-70.74)
L2N	85	15,000	51.52 (41.15-63.70)
V4A	90	16,000	51.14 (41.12-62.86)
V8S	45	8,000	51.14 (37.30-68.42)
S9H	45	8,000	51.14 (37.30-68.42)
S0E	95	17,000	50.80 (41.10-62.10)

R3M	55	10,000	50.00 (37.67-65.08)
L8V	55	10,000	50.00 (37.67-65.08)
V8V	55	10,000	50.00 (37.67-65.08)
S0N	60	11,000	49.59 (37.84-63.83)
H4W	60	11,000	49.59 (37.84-63.83)
T5R	65	12,000	49.24 (38.00-62.76)
K7M	110	21,000	47.62 (39.14-57.39)
S0G	165	32,000	46.88 (40.00-54.60)
L3V	100	20,000	45.45 (36.98-55.28)
V2P	80	16,000	45.45 (36.04-56.57)
V1T	75	15,000	45.45 (35.75-56.98)
K8N	65	13,000	45.45 (35.08-57.94)
K8A	60	12,000	45.45 (34.69-58.51)
H9S	50	10,000	45.45 (33.74-59.93)
V4T	50	10,000	45.45 (33.74-59.93)
H7V	50	10,000	45.45 (33.74-59.93)
H8P	50	10,000	45.45 (33.74-59.93)
L2E	50	10,000	45.45 (33.74-59.93)
R3L	45	9,000	45.45 (33.15-60.82)
L9Y	45	9,000	45.45 (33.15-60.82)
V4B	40	8,000	45.45 (32.47-61.90)
L2A	40	8,000	45.45 (32.47-61.90)
L4R	40	8,000	45.45 (32.47-61.90)

R3P	40	8,000	45.45 (32.47-61.90)
A0N	35	7,000	45.45 (31.66-63.22)
L1A	35	7,000	45.45 (31.66-63.22)
T6A	35	7,000	45.45 (31.66-63.22)
L8M	35	7,000	45.45 (31.66-63.22)
E4P	30	6,000	45.45 (30.67-64.89)
P2A	30	6,000	45.45 (30.67-64.89)
V1R	25	5,000	45.45 (29.42-67.10)
J4P	25	5,000	45.45 (29.42-67.10)
T4J	25	5,000	45.45 (29.42-67.10)
V8K	25	5,000	45.45 (29.42-67.10)
L7S	25	5,000	45.45 (29.42-67.10)
S0K	180	37,000	44.23 (38.00-51.18)
N3R	75	16,000	42.61 (33.52-53.42)
B0E	70	15,000	42.42 (33.07-53.60)
J2S	60	13,000	41.96 (32.02-54.01)
K6V	60	13,000	41.96 (32.02-54.01)
N7T	60	13,000	41.96 (32.02-54.01)
P3A	55	12,000	41.67 (31.39-54.23)
S0C	50	11,000	41.32 (30.67-54.48)
T0B	140	31,000	41.06 (34.54-48.45)
T5K	45	10,000	40.91 (29.84-54.74)
V8R	45	10,000	40.91 (29.84-54.74)

G6G	45	10,000	40.91 (29.84-54.74)
V0X	45	10,000	40.91 (29.84-54.74)
N8Y	40	9,000	40.40 (28.87-55.02)
B0M	40	9,000	40.40 (28.87-55.02)
V8A	40	9,000	40.40 (28.87-55.02)
T0K	115	26,000	40.21 (33.20-48.27)
K0C	110	25,000	40.00 (32.88-48.21)
B0T	35	8,000	39.77 (27.70-55.31)
R2X	35	8,000	39.77 (27.70-55.31)
A1B	35	8,000	39.77 (27.70-55.31)
K2B	65	15,000	39.39 (30.40-50.21)
L9H	65	15,000	39.39 (30.40-50.21)
S7J	65	15,000	39.39 (30.40-50.21)
N6H	65	15,000	39.39 (30.40-50.21)
R2G	65	15,000	39.39 (30.40-50.21)
N0H	95	22,000	39.26 (31.76-47.99)
C1A	60	14,000	38.96 (29.73-50.15)
B1P	30	7,000	38.96 (26.29-55.62)
B2H	30	7,000	38.96 (26.29-55.62)
J4H	30	7,000	38.96 (26.29-55.62)
K7R	30	7,000	38.96 (26.29-55.62)
V8M	30	7,000	38.96 (26.29-55.62)
L8S	30	7,000	38.96 (26.29-55.62)

B2Y	30	7,000	38.96 (26.29-55.62)
K7H	30	7,000	38.96 (26.29-55.62)
R2H	30	7,000	38.96 (26.29-55.62)
V4M	30	7,000	38.96 (26.29-55.62)
H1T	55	13,000	38.46 (28.97-50.06)
P7A	55	13,000	38.46 (28.97-50.06)
R0K	55	13,000	38.46 (28.97-50.06)
R0C	105	25,000	38.18 (31.23-46.22)
L0S	100	24,000	37.88 (30.82-46.07)
J6E	75	18,000	37.88 (29.79-47.48)
J2C	50	12,000	37.88 (28.11-49.94)
K9H	50	12,000	37.88 (28.11-49.94)
B5A	25	6,000	37.88 (24.51-55.92)
L7N	25	6,000	37.88 (24.51-55.92)
G8Z	25	6,000	37.88 (24.51-55.92)
S4P	25	6,000	37.88 (24.51-55.92)
R3B	25	6,000	37.88 (24.51-55.92)
R3K	25	6,000	37.88 (24.51-55.92)
N1A	25	6,000	37.88 (24.51-55.92)
M4A	25	6,000	37.88 (24.51-55.92)
N3L	25	6,000	37.88 (24.51-55.92)
N7A	25	6,000	37.88 (24.51-55.92)
S0M	95	23,000	37.55 (30.38-45.90)

K0K	210	51,000	37.43 (32.54-42.85)
K0L	140	34,000	37.43 (31.49-44.17)
V2S	90	22,000	37.19 (29.91-45.71)
V3L	45	11,000	37.19 (27.13-49.76)
L0K	65	16,000	36.93 (28.50-47.07)
L2M	65	16,000	36.93 (28.50-47.07)
T2V	65	16,000	36.93 (28.50-47.07)
V1X	60	15,000	36.36 (27.75-46.81)
G9A	40	10,000	36.36 (25.98-49.52)
L0E	40	10,000	36.36 (25.98-49.52)
B0K	75	19,000	35.89 (28.23-44.98)
N0M	125	32,000	35.51 (29.56-42.31)
G0L	125	32,000	35.51 (29.56-42.31)
N0G	160	41,000	35.48 (30.19-41.42)
E2M	35	9,000	35.35 (24.63-49.17)
L3K	35	9,000	35.35 (24.63-49.17)
M9P	35	9,000	35.35 (24.63-49.17)
V5Z	35	9,000	35.35 (24.63-49.17)
R7B	35	9,000	35.35 (24.63-49.17)
B3H	35	9,000	35.35 (24.63-49.17)
N1S	35	9,000	35.35 (24.63-49.17)
P0P	35	9,000	35.35 (24.63-49.17)
J0X	85	22,000	35.12 (28.06-43.43)

N7M	50	13,000	34.97 (25.95-46.10)
V0G	50	13,000	34.97 (25.95-46.10)
M9B	50	13,000	34.97 (25.95-46.10)
T1A	50	13,000	34.97 (25.95-46.10)
K9J	80	21,000	34.63 (27.46-43.10)
R0E	75	20,000	34.09 (26.81-42.73)
G6P	60	16,000	34.09 (26.01-43.88)
P1B	60	16,000	34.09 (26.01-43.88)
V1W	45	12,000	34.09 (24.87-45.62)
L2G	45	12,000	34.09 (24.87-45.62)
L2R	45	12,000	34.09 (24.87-45.62)
N7L	45	12,000	34.09 (24.87-45.62)
K0M	85	23,000	33.60 (26.84-41.54)
V3A	70	19,000	33.49 (26.11-42.32)
C0B	55	15,000	33.33 (25.11-43.39)
N0A	55	15,000	33.33 (25.11-43.39)
M2R	65	18,000	32.83 (25.34-41.84)
V0R	115	32,000	32.67 (26.97-39.22)
K0H	75	21,000	32.47 (25.54-40.70)
R0G	75	21,000	32.47 (25.54-40.70)
K6H	50	14,000	32.47 (24.10-42.80)
P6A	60	17,000	32.09 (24.48-41.30)
H1G	80	23,000	31.62 (25.07-39.35)

G0R	140	42,000	30.30 (25.49-35.76)

† Number of deaths was rounded to a multiple of 5 as per SSHRC/Statistics Canada regulations.

Supplementary Table 7. Overall prostate cancer mortality rates by Forward Sortation Area (FSA) between 1992 and 2010. Mortality rates are expressed per 100,000 men per year. Only FSAs with statistically significant lower mortality rates compared to the national average are included in this table.

**FSA**	**Cases †**	**Male Population (rounded to 1,000)**	**Incidence Rate (95% CI)**
K0A	95	46,000	18.77 (15.19-22.95)
J0L	80	39,000	18.65 (14.79-23.21)
P0M	50	26,000	17.48 (12.98-23.05)
L4L	50	26,000	17.48 (12.98-23.05)
V0J	55	29,000	17.24 (12.99-22.44)
G0X	60	32,000	17.05 (13.01-21.94)
M2N	45	24,000	17.05 (12.43-22.81)
L1S	35	19,000	16.75 (11.66-23.29)
V3J	35	19,000	16.75 (11.66-23.29)
L0L	45	25,000	16.36 (11.94-21.90)
L6H	45	25,000	16.36 (11.94-21.90)
T5T	45	25,000	16.36 (11.94-21.90)
M1S	30	17,000	16.04 (10.82-22.90)
M3C	30	17,000	16.04 (10.82-22.90)
T8V	30	17,000	16.04 (10.82-22.90)
T5X	30	17,000	16.04 (10.82-22.90)
V4C	35	20,000	15.91 (11.08-22.13)

L0G	35	20,000	15.91 (11.08-22.13)
M2J	45	26,000	15.73 (11.48-21.05)
G0A	80	48,000	15.15 (12.01-18.86)
N0R	35	21,000	15.15 (10.55-21.07)
G0W	35	21,000	15.15 (10.55-21.07)
J0Y	30	18,000	15.15 (10.22-21.63)
H7W	30	18,000	15.15 (10.22-21.63)
V3C	30	18,000	15.15 (10.22-21.63)
T2K	30	18,000	15.15 (10.22-21.63)
H7N	30	18,000	15.15 (10.22-21.63)
M8V	25	15,000	15.15 (9.81-22.37)
M4K	25	15,000	15.15 (9.81-22.37)
N5Y	25	15,000	15.15 (9.81-22.37)
L5C	25	15,000	15.15 (9.81-22.37)
M3J	20	12,000	15.15 (9.25-23.40)
R3G	20	12,000	15.15 (9.25-23.40)
L9S	20	12,000	15.15 (9.25-23.40)
N5W	20	12,000	15.15 (9.25-23.40)
R2P	20	12,000	15.15 (9.25-23.40)
H4N	20	12,000	15.15 (9.25-23.40)
J4K	20	12,000	15.15 (9.25-23.40)
L4P	20	12,000	15.15 (9.25-23.40)
V7A	20	12,000	15.15 (9.25-23.40)

J0H	45	28,000	14.61 (10.66-19.55)
J7V	45	28,000	14.61 (10.66-19.55)
L1J	35	22,000	14.46 (10.07-20.11)
M9W	30	19,000	14.35 (9.68-20.49)
M6E	30	19,000	14.35 (9.68-20.49)
M6M	30	19,000	14.35 (9.68-20.49)
J3L	30	19,000	14.35 (9.68-20.49)
L6X	30	19,000	14.35 (9.68-20.49)
G1C	25	16,000	14.20 (9.19-20.97)
L6S	40	26,000	13.99 (9.99-19.04)
H2S	20	13,000	13.99 (8.54-21.60)
T5H	20	13,000	13.99 (8.54-21.60)
T2T	20	13,000	13.99 (8.54-21.60)
T2X	20	13,000	13.99 (8.54-21.60)
M6H	35	23,000	13.83 (9.64-19.24)
L3T	35	23,000	13.83 (9.64-19.24)
L5B	35	23,000	13.83 (9.64-19.24)
M1B	45	30,000	13.64 (9.95-18.25)
J0A	15	10,000	13.64 (7.63-22.49)
V2G	15	10,000	13.64 (7.63-22.49)
V8G	15	10,000	13.64 (7.63-22.49)
H1V	15	10,000	13.64 (7.63-22.49)
L4X	15	10,000	13.64 (7.63-22.49)

L5G	15	10,000	13.64 (7.63-22.49)
T4B	15	10,000	13.64 (7.63-22.49)
H2R	15	10,000	13.64 (7.63-22.49)
P0V	15	10,000	13.64 (7.63-22.49)
G7S	15	10,000	13.64 (7.63-22.49)
K1R	15	10,000	13.64 (7.63-22.49)
T3L	15	10,000	13.64 (7.63-22.49)
V2K	15	10,000	13.64 (7.63-22.49)
H1Z	25	17,000	13.37 (8.65-19.74)
J5R	25	17,000	13.37 (8.65-19.74)
M1C	25	17,000	13.37 (8.65-19.74)
L8E	25	17,000	13.37 (8.65-19.74)
T3A	35	24,000	13.26 (9.23-18.44)
L3R	40	28,000	12.99 (9.28-17.68)
V3V	30	21,000	12.99 (8.76-18.54)
H2V	20	14,000	12.99 (7.93-20.06)
H1R	20	14,000	12.99 (7.93-20.06)
J9X	20	14,000	12.99 (7.93-20.06)
N6E	20	14,000	12.99 (7.93-20.06)
V3E	20	14,000	12.99 (7.93-20.06)
V3R	35	25,000	12.73 (8.87-17.70)
J4B	25	18,000	12.63 (8.17-18.64)
T2W	30	22,000	12.40 (8.36-17.70)

G1G	15	11,000	12.40 (6.94-20.45)
J3H	15	11,000	12.40 (6.94-20.45)
T5W	15	11,000	12.40 (6.94-20.45)
V5A	15	11,000	12.40 (6.94-20.45)
M5M	15	11,000	12.40 (6.94-20.45)
N3C	15	11,000	12.40 (6.94-20.45)
G2B	15	11,000	12.40 (6.94-20.45)
N2K	15	11,000	12.40 (6.94-20.45)
V1J	15	11,000	12.40 (6.94-20.45)
G2E	15	11,000	12.40 (6.94-20.45)
J7K	15	11,000	12.40 (6.94-20.45)
J7P	15	11,000	12.40 (6.94-20.45)
T6L	35	26,000	12.24 (8.52-17.02)
L6Y	40	30,000	12.12 (8.66-16.51)
H7L	20	15,000	12.12 (7.40-18.72)
J4J	20	15,000	12.12 (7.40-18.72)
M4L	20	15,000	12.12 (7.40-18.72)
T6K	20	15,000	12.12 (7.40-18.72)
V3W	50	38,000	11.96 (8.88-15.77)
J8T	25	19,000	11.96 (7.74-17.66)
L6T	25	19,000	11.96 (7.74-17.66)
K1C	25	19,000	11.96 (7.74-17.66)
L6M	25	19,000	11.96 (7.74-17.66)

J3Y	35	28,000	11.36 (7.92-15.80)
M9V	35	28,000	11.36 (7.92-15.80)
J0R	30	24,000	11.36 (7.67-16.22)
T8A	25	20,000	11.36 (7.35-16.77)
M6N	25	20,000	11.36 (7.35-16.77)
L4T	25	20,000	11.36 (7.35-16.77)
V5R	25	20,000	11.36 (7.35-16.77)
H3S	20	16,000	11.36 (6.94-17.55)
V2N	20	16,000	11.36 (6.94-17.55)
M3M	15	12,000	11.36 (6.36-18.74)
N2N	15	12,000	11.36 (6.36-18.74)
V5C	15	12,000	11.36 (6.36-18.74)
A1N	15	12,000	11.36 (6.36-18.74)
N5Z	15	12,000	11.36 (6.36-18.74)
X0E	15	12,000	11.36 (6.36-18.74)
T1S	15	12,000	11.36 (6.36-18.74)
J1N	15	12,000	11.36 (6.36-18.74)
J5Z	15	12,000	11.36 (6.36-18.74)
K1T	15	12,000	11.36 (6.36-18.74)
T6R	15	12,000	11.36 (6.36-18.74)
G1P	10	8,000	11.36 (5.45-20.90)
V3X	10	8,000	11.36 (5.45-20.90)
J5M	10	8,000	11.36 (5.45-20.90)

E2E	10	8,000	11.36 (5.45-20.90)
V3Y	10	8,000	11.36 (5.45-20.90)
L1W	10	8,000	11.36 (5.45-20.90)
V9C	10	8,000	11.36 (5.45-20.90)
J4Y	10	8,000	11.36 (5.45-20.90)
K1E	10	8,000	11.36 (5.45-20.90)
T9M	10	8,000	11.36 (5.45-20.90)
V9Z	10	8,000	11.36 (5.45-20.90)
N9J	10	8,000	11.36 (5.45-20.90)
T2A	35	29,000	10.97 (7.64-15.26)
L4G	25	21,000	10.82 (7.00-15.98)
R0B	25	21,000	10.82 (7.00-15.98)
V4N	25	21,000	10.82 (7.00-15.98)
L8L	20	17,000	10.70 (6.53-16.52)
M1J	20	17,000	10.70 (6.53-16.52)
V7E	20	17,000	10.70 (6.53-16.52)
L7M	20	17,000	10.70 (6.53-16.52)
M5A	20	17,000	10.70 (6.53-16.52)
J9H	15	13,000	10.49 (5.87-17.30)
H7M	15	13,000	10.49 (5.87-17.30)
H2J	15	13,000	10.49 (5.87-17.30)
T1Y	30	27,000	10.10 (6.82-14.42)
T2Y	20	18,000	10.10 (6.17-15.60)

J4W	10	9,000	10.10 (4.84-18.58)
J6Z	10	9,000	10.10 (4.84-18.58)
H7K	10	9,000	10.10 (4.84-18.58)
L8J	10	9,000	10.10 (4.84-18.58)
V3G	10	9,000	10.10 (4.84-18.58)
J4T	10	9,000	10.10 (4.84-18.58)
K1B	10	9,000	10.10 (4.84-18.58)
T5Z	10	9,000	10.10 (4.84-18.58)
G0V	10	9,000	10.10 (4.84-18.58)
J0M	10	9,000	10.10 (4.84-18.58)
R2R	10	9,000	10.10 (4.84-18.58)
L5L	25	23,000	9.88 (6.39-14.59)
L7E	15	14,000	9.74 (5.45-16.07)
K2J	20	19,000	9.57 (5.85-14.78)
L5M	35	34,000	9.36 (6.52-13.02)
L6V	20	20,000	9.09 (5.55-14.04)
J4L	15	15,000	9.09 (5.09-14.99)
G8Y	15	15,000	9.09 (5.09-14.99)
N6G	15	15,000	9.09 (5.09-14.99)
L4Z	15	15,000	9.09 (5.09-14.99)
J4Z	10	10,000	9.09 (4.36-16.72)
J7T	10	10,000	9.09 (4.36-16.72)
K1Z	10	10,000	9.09 (4.36-16.72)

K2L	10	10,000	9.09 (4.36-16.72)
M9M	10	10,000	9.09 (4.36-16.72)
V5L	10	10,000	9.09 (4.36-16.72)
S4X	10	10,000	9.09 (4.36-16.72)
B4C	10	10,000	9.09 (4.36-16.72)
J3X	10	10,000	9.09 (4.36-16.72)
M4X	10	10,000	9.09 (4.36-16.72)
L9N	5	5,000	9.09 (2.95-21.22)
V7G	5	5,000	9.09 (2.95-21.22)
B3G	5	5,000	9.09 (2.95-21.22)
G4Z	5	5,000	9.09 (2.95-21.22)
G8K	5	5,000	9.09 (2.95-21.22)
H3Y	5	5,000	9.09 (2.95-21.22)
N2V	5	5,000	9.09 (2.95-21.22)
V2W	5	5,000	9.09 (2.95-21.22)
J2R	5	5,000	9.09 (2.95-21.22)
J7L	5	5,000	9.09 (2.95-21.22)
P0S	5	5,000	9.09 (2.95-21.22)
L4J	30	31,000	8.80 (5.94-12.56)
K2G	20	21,000	8.66 (5.29-13.37)
T3G	20	21,000	8.66 (5.29-13.37)
V5N	15	16,000	8.52 (4.77-14.06)
H3N	15	16,000	8.52 (4.77-14.06)

J7C	15	16,000	8.52 (4.77-14.06)
M1V	25	27,000	8.42 (5.45-12.43)
L5N	35	38,000	8.37 (5.83-11.65)
J6X	10	11,000	8.26 (3.96-15.20)
L3Z	10	11,000	8.26 (3.96-15.20)
V5T	10	11,000	8.26 (3.96-15.20)
H9J	10	11,000	8.26 (3.96-15.20)
L1E	10	11,000	8.26 (3.96-15.20)
T4P	10	11,000	8.26 (3.96-15.20)
L1K	10	11,000	8.26 (3.96-15.20)
V5V	10	11,000	8.26 (3.96-15.20)
J2W	10	11,000	8.26 (3.96-15.20)
J5Y	10	11,000	8.26 (3.96-15.20)
V6X	10	11,000	8.26 (3.96-15.20)
Y1A	10	11,000	8.26 (3.96-15.20)
L6A	20	23,000	7.91 (4.83-12.21)
J0N	20	23,000	7.91 (4.83-12.21)
T2B	10	12,000	7.58 (3.63-13.93)
L4S	10	12,000	7.58 (3.63-13.93)
J3E	10	12,000	7.58 (3.63-13.93)
L4E	10	12,000	7.58 (3.63-13.93)
L6C	10	12,000	7.58 (3.63-13.93)
J5A	10	12,000	7.58 (3.63-13.93)

A0P	5	6,000	7.58 (2.46-17.68)
G6E	5	6,000	7.58 (2.46-17.68)
J2J	5	6,000	7.58 (2.46-17.68)
T7S	5	6,000	7.58 (2.46-17.68)
B6L	5	6,000	7.58 (2.46-17.68)
H2W	5	6,000	7.58 (2.46-17.68)
T6T	5	6,000	7.58 (2.46-17.68)
H1C	5	6,000	7.58 (2.46-17.68)
H4M	5	6,000	7.58 (2.46-17.68)
J5J	5	6,000	7.58 (2.46-17.68)
V4E	5	6,000	7.58 (2.46-17.68)
V5Y	5	6,000	7.58 (2.46-17.68)
L3X	10	13,000	6.99 (3.35-12.86)
T5Y	10	13,000	6.99 (3.35-12.86)
M3N	15	21,000	6.49 (3.63-10.71)
L1R	10	14,000	6.49 (3.11-11.94)
G0B	5	7,000	6.49 (2.11-15.15)
H8Z	5	7,000	6.49 (2.11-15.15)
N2P	5	7,000	6.49 (2.11-15.15)
J8Z	5	7,000	6.49 (2.11-15.15)
T2P	5	7,000	6.49 (2.11-15.15)
G3K	5	7,000	6.49 (2.11-15.15)
J9A	5	7,000	6.49 (2.11-15.15)

L4B	10	15,000	6.06 (2.91-11.15)
L7A	10	15,000	6.06 (2.91-11.15)
H7P	10	15,000	6.06 (2.91-11.15)
N2E	10	15,000	6.06 (2.91-11.15)
K4A	10	15,000	6.06 (2.91-11.15)
L3S	15	23,000	5.93 (3.32-9.78)
L5R	10	16,000	5.68 (2.72-10.45)
L6Z	10	16,000	5.68 (2.72-10.45)
G7A	5	8,000	5.68 (1.84-13.26)
M4P	5	8,000	5.68 (1.84-13.26)
L6E	5	8,000	5.68 (1.84-13.26)
H2T	5	8,000	5.68 (1.84-13.26)
H9A	5	8,000	5.68 (1.84-13.26)
N2T	5	8,000	5.68 (1.84-13.26)
T3K	15	26,000	5.24 (2.94-8.65)
T2Z	10	18,000	5.05 (2.42-9.29)
G6Z	5	9,000	5.05 (1.64-11.79)
L5V	10	19,000	4.78 (2.29-8.80)
J7M	5	10,000	4.55 (1.48-10.61)
T2C	5	10,000	4.55 (1.48-10.61)
J8V	5	10,000	4.55 (1.48-10.61)
J8R	5	11,000	4.13 (1.34-9.64)
L6R	10	23,000	3.95 (1.90-7.27)

T3J	10	23,000	3.95 (1.90-7.27)
L4H	5	15,000	3.03 (0.98-7.07)
L6P	5	16,000	2.84 (0.92-6.63)
L1T	5	17,000	2.67 (0.87-6.24)

† Number of deaths was rounded to a multiple of 5 as per SSHRC/Statistics Canada regulations.

Supplementary Table 8. Percentage of population in each Canadian province that is considered obese. Percentage of population that is > the age of 65. Percentage of population that identify themselves as Black ethnicity.

Province	Percentage of population that is obese* (%)	Percentage of population that is > 65** (%)	Percentage of population of Black ethnicity** (%)
Quebec	22.80	13.90	2.34
Alberta	25.90	10.50	1.27
Newfoundland and Labrador	35.20	13.30	0.17
Ontario	24.90	13.40	3.80
Manitoba	27.70	14.00	1.27
Saskatchewan	31.60	15.00	0.48
British Columbia	20.40	13.60	0.68
Nova Scotia	32.30	14.70	2.16
New Brunswick	33.20	14.40	0.58
Prince Edward Island	32.40	14.50	0.38
Nunavut	33.00	2.60	0.29
Northwest Territories	35.30	4.50	0.70
Yukon	26.80	6.80	0.42

* Source: Canadian Community Health Survey, 2011–2012.

**Source: Canadian Census of population.

Supplementary Table 9. Incidence rate ratio (IRR) analyses for each FSA socioeconomic status quintile. The IRRs compare the given quintile to Quintile 1 (lowest socioeconomic status). An IRR >1 for a given quintile represents an incidence rate that is higher than that of Quintile 1. An IRR <1 for a given quintile represents an incidence rate that is lower than that of Quintile 1.

Quintile	Average median income range* (CAD)	Total number of cases	Total male population (rounded to 1,000)	IRR (95% CI)
Quintile 1	<20,000	93,955	4,434,000	-
Quintile 2	20,000 – 25,000	117,695	5,260,000	1.06 (1.05-1.07)
Quintile 3	25,000 – 30,000	64,715	3,190,000	0.96 (0.95-0.97)
Quintile 4	30,000 – 35,000	18,475	1,040,000	0.84 (0.83-0.85)
Quintile 5	>35,000	4,865	289,000	0.79 (0.77-0.82)

* Source: Canadian Census of Population, 2001 and 2006

Supplementary Table 10. Incidence rate ratio (IRR) analyses for each FSA Black visible minority quintile. The IRRs compare the given quintile to Quintile 1 (lowest percentage of Black individuals). An IRR >1 for a given quintile represents an incidence rate that is higher than that of Quintile 1. An IRR <1 for a given quintile represents an incidence rate that is lower than that of Quintile 1.

**Quintile**	**Average percentage of Black individuals* (%)**	**Total number of cases**	**Total male population (rounded to 1,000)**	**IRR (95% CI)**
Quintile 1	0.00-1.99	220,355	9,610,000	-
Quintile 2	2.00-3.99	65,385	2,223,000	1.28 (1.27-1.29)
Quintile 3	4.00-5.99	13,415	798,000	0.73 (0.72-0.75)
Quintile 4	6.00-7.99	8,280	494,000	0.73 (0.72-0.75)
Quintile 5	≥8.00	18,590	1,113,000	0.73 (0.72-0.74)

* Source: Canadian Census of Population, 2001 and 2006

Supplementary Table 11. Analyses of ratios of mortality rates for each FSA Black visible minority quintile. The ratios compare the given quintile to Quintile 1 (lowest percentage of Black individuals). A ratio >1 for a given quintile represents a mortality rate that is higher than that of Quintile 1. A ratio <1 for a given quintile represents a mortality rate that is lower than that of Quintile 1.

Quintile	Average percentage of Black individuals* (%)	Total number of deaths	Total male population (rounded to 1,000)	Ratio of mortality rates (95% CI)
Quintile 1	0.00-1.99	26,380	9,610,000	-
Quintile 2	2.00-3.99	7,680	2,223,000	1.26 (1.23-1.29)
Quintile 3	4.00-5.99	1,550	798,000	0.71 (0.67-0.74)
Quintile 4	6.00-7.99	845	494,000	0.62 (0.58-0.67)
Quintile 5	≥8.00	1,990	1,113,000	0.65 (0.62-0.68)

* Source: Canadian Census of Population, 2001 and 2006

Supplementary Table 12. Provincial mortality rates (per 100,000 men per year) of all deaths by prostate cancer between 1992 and 2010 with linear regression analysis of the mortality rate over time. The slope of the linear regression analysis represents the overall annual change in mortality rates and is expressed as deaths per 100,000 men per year.

Year	Deaths	Crude mortality rate per 100,000 men	Slope of linear regression analysis (95% CI)	P-value
**Newfoundland and Labrador**				
1992	65	22.34	0.27 (-0.15 – 0.68)	0.19
1993	60	20.62		
1994	60	20.91		
1995	70	24.73		
1996	45	16.13		
1997	75	27.37		
1998	60	22.47		
1999	75	28.41		
2000	70	26.82		
2001	100	38.91		
2002	60	23.44		
2003	65	25.39		
2004	75	29.41		
2005	60	23.62		
2006	75	29.76		
2007	65	26.00		
2008	70	27.78		
2009	65	25.59		
2010	55	21.40		
**Prince Edward Island**				
1992	30	46.15	-0.62 (-1.25 – 0.02)	0.06
1993	20	30.77		
1994	30	45.45		
1995	25	37.88		
1996	25	37.31		
1997	20	29.85		
1998	15	22.39		
1999	20	29.85		
2000	20	29.85		

2001	25	37.31		
2002	20	29.85		
2003	15	22.39		
2004	30	44.78		
2005	15	22.06		
2006	20	29.41		
2007	20	29.85		
2008	20	29.41		
2009	25	36.76		
2010	15	21.74		
**Nova Scotia**				
1992	150	33.11	-0.29 (-0.47 – -0.10)	<0.005
1993	130	28.57		
1994	150	32.89		
1995	140	30.70		
1996	150	32.82		
1997	140	30.63		
1998	150	32.82		
1999	135	29.48		
2000	145	31.73		
2001	135	29.61		
2002	130	28.38		
2003	105	22.93		
2004	125	27.23		
2005	130	28.45		
2006	130	28.51		
2007	120	26.37		
2008	130	28.51		
2009	125	27.29		
2010	135	29.28		
**New Brunswick**				
1992	105	28.30	-0.17 (-0.51 – 0.16)	0.30
1993	105	28.30		
1994	105	28.23		
1995	100	26.88		
1996	95	25.47		
1997	105	28.15		
1998	135	36.29		
1999	100	26.95		
2000	100	26.95		
2001	125	33.69		
2002	105	28.38		

2003	125	33.88		
2004	90	24.39		
2005	105	28.53		
2006	80	21.86		
2007	105	28.69		
2008	115	31.25		
2009	90	24.32		
2010	80	21.51		
**Quebec**				
1992	775	22.14	-0.21 (-0.29 – -0.13)	<0.001
1993	805	22.84		
1994	790	22.30		
1995	850	23.91		
1996	765	21.44		
1997	770	21.48		
1998	765	21.29		
1999	785	21.76		
2000	820	22.61		
2001	805	22.07		
2002	795	21.64		
2003	770	20.82		
2004	735	19.73		
2005	755	20.13		
2006	705	18.66		
2007	700	18.38		
2008	730	18.99		
2009	775	19.94		
2010	805	20.47		
**Ontario**				
1992	1,265	24.23	-0.20 (-0.25 – -0.14)	<0.001
1993	1,320	25.02		
1994	1,350	25.31		
1995	1,365	25.30		
1996	1,335	24.46		
1997	1,320	23.84		
1998	1,350	24.09		
1999	1,320	23.26		
2000	1,335	23.14		
2001	1,375	23.39		
2002	1,385	23.17		
2003	1,370	22.64		
2004	1,375	22.46		

2005	1,295	20.92		
2006	1,385	22.15		
2007	1,350	21.46		
2008	1,415	22.31		
2009	1,400	21.91		
2010	1,470	22.78		
**Manitoba**				
1992	180	32.61	-0.13 (-0.33 – 0.06)	0.17
1993	175	31.59		
1994	165	29.62		
1995	180	32.14		
1996	165	29.36		
1997	170	30.20		
1998	190	33.69		
1999	150	26.46		
2000	180	31.63		
2001	175	30.65		
2002	170	29.62		
2003	175	30.33		
2004	185	31.73		
2005	185	31.62		
2006	140	23.81		
2007	190	32.15		
2008	175	29.41		
2009	170	28.33		
2010	180	29.70		
**Saskatchewan**				
1992	185	37.00	-0.14 (-0.48 – 0.19)	0.38
1993	185	36.93		
1994	205	40.84		
1995	200	39.68		
1996	200	39.45		
1997	225	44.47		
1998	220	43.48		
1999	235	46.53		
2000	230	45.91		
2001	215	43.26		
2002	215	43.43		
2003	225	45.45		
2004	215	43.43		
2005	185	37.53		
2006	205	41.67		

2007	195	39.24		
2008	200	39.53		
2009	175	33.91		
2010	180	34.22		
**Alberta**				
1992	290	21.87	-0.18 (-0.29 – -0.07)	<0.005
1993	290	21.61		
1994	290	21.34		
1995	340	24.71		
1996	310	22.19		
1997	320	22.44		
1998	335	22.90		
1999	280	18.78		
2000	345	22.74		
2001	330	21.35		
2002	345	21.79		
2003	360	22.35		
2004	335	20.43		
2005	345	20.50		
2006	350	20.15		
2007	340	19.03		
2008	360	19.67		
2009	365	19.50		
2010	370	19.53		
**British Columbia**				
1992	450	26.04	-0.12 (-0.25 – 0.00)	<0.05
1993	490	27.57		
1994	475	25.93		
1995	485	25.74		
1996	490	25.40		
1997	475	24.16		
1998	450	22.72		
1999	505	25.34		
2000	475	23.69		
2001	540	26.69		
2002	490	24.10		
2003	440	21.52		
2004	520	25.25		
2005	505	24.29		
2006	470	22.37		
2007	540	25.38		
2008	500	23.16		

2009	550	25.10		
2010	540	24.34		
